# Cellular senescence escape and antiviral response discriminate glioblastoma from lower-grade gliomas

**DOI:** 10.1093/noajnl/vdag122

**Published:** 2026-04-08

**Authors:** Dominika Maurencova, Michaela Sadibolova, Monika Zarska, Zuzana Liblova, Josef Novak, Jirina Kroupova, Pavla Vasicova, Jiri Bartek, Zdenek Hodny

**Affiliations:** Laboratory of Genome Integrity, Institute of Molecular Genetics of the CAS, Prague, Czech Republic; Biomedical Research Centre, University Hospital Hradec Kralove, Hradec Kralove, Czech Republic; Laboratory of Genome Integrity, Institute of Molecular Genetics of the CAS, Prague, Czech Republic; Laboratory of Genome Integrity, Institute of Molecular Genetics of the CAS, Prague, Czech Republic; Laboratory of Genome Integrity, Institute of Molecular Genetics of the CAS, Prague, Czech Republic; Laboratory of Genome Integrity, Institute of Molecular Genetics of the CAS, Prague, Czech Republic; Laboratory of Genome Integrity, Institute of Molecular Genetics of the CAS, Prague, Czech Republic; Laboratory of Genome Integrity, Institute of Molecular Genetics of the CAS, Prague, Czech Republic; Danish Cancer Institute, Copenhagen, Denmark (J.B.); Division of Genome Biology, Department of Medical Biochemistry and Biophysics, Science of Life Laboratory, Karolinska Institutet, Stockholm, Sweden; Laboratory of Genome Integrity, Institute of Molecular Genetics of the CAS, Prague, Czech Republic

**Keywords:** antiviral response, cellular senescence, glioblastoma, multi‑omics

## Abstract

***Background.*** Cellular senescence is a negative prognostic indicator in glioblastoma (GB). However, the specific cell types exhibiting senescence and the molecular mechanisms by which senescent cells (SCs) contribute to GB pathogenesis remain unknown.

***Methods.*** We performed multi-omics integration of publicly available GB patient-derived datasets to identify SCs and their functional impact on GB pathogenesis. We created a transcriptomic definition of SCs to verify their presence in GB datasets. Next, we analyzed transcriptomic profiles of GB and low-grade gliomas to reveal key features of GB aggressiveness. The GB patients’ phosphoproteome was analyzed with a focus on key regulators of senescence. To assess chemotherapy-evoked secondary senescence, we performed a proteomic analysis of temozolomide-induced senescence in human GB spheroids.

***Results.*** We identified GB-associated SCs in clusters radial glia, endothelial cells, and immature astrocytes localized primarily in the hypoxic zones. Notably, we identified senescence-escape features and tumor antiviral responses as processes distinguishing GB from low-grade gliomas. In GB samples, we detected inhibitory phosphorylation of p21 and p27 proteins and active PI3K signaling, which can lead to senescence escape and belong to the typical manipulation arsenal of herpesviruses. Our proteomic analysis of temozolomide-induced senescent GB cells reveals that primary (pre-radiochemotherapy) and secondary senescence in GB share similar phenotypic features. Pathways associated with GB aggressiveness are upregulated after therapy, which can promote more aggressive behavior of recurrent tumors.

***Conclusions.*** Our data indicate that senescence and viral reactivation may fuel GB progression, including recurrence, suggesting that senolytics and antiviral drugs are potential therapeutic avenues.

## Graphical Abstract

**Graphical Abstract:**
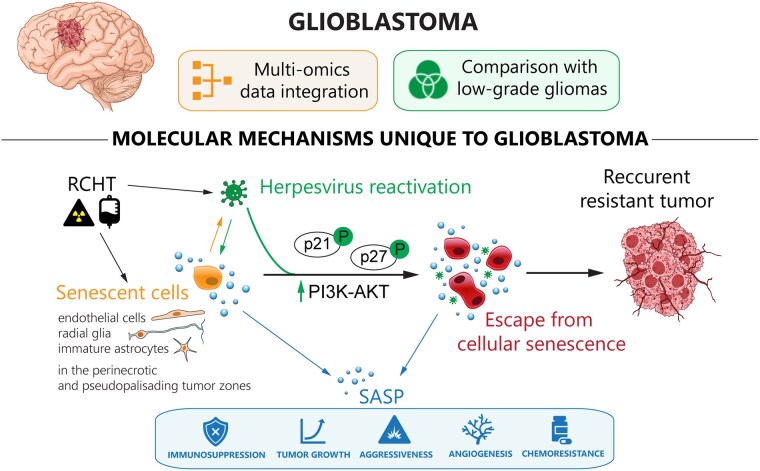

Key Points

- Identification of senescent cells in glioblastoma patient samples.
- Senescence escape and antiviral response differentiate glioblastoma from low-grade gliomas.
- Therapy-induced senescent cells can contribute to recurrent glioblastoma formation.

Importance of the Study

Over the past decades, therapeutic advancements have yielded only marginal improvements in glioblastoma (GB) patient outcomes, with overall survival remaining limited. Senescent cells (SCs) promote the malignant progression of GB and negatively affect survival. However, the molecular mechanisms by which they contribute to GB pathogenesis remain poorly understood. Evaluating the role of SCs in human diseases, including cancer, requires a multifaceted approach. Our integrative multi-omics strategy confirmed the presence of SCs in GB patient samples, moreover, it revealed cells escaping senescence and active herpesvirus reactivation as possible drivers of GB aggressiveness. At the same time, the data suggests that both processes, senescence escape and viral infection/reactivation, could be functionally linked. Our data confirms that primary and secondary temozolomide-induced SCs share similar features. Thus, GB-associated SCs are ideal candidates for the development of more aggressive recurrent tumors. Overall, our integrative approach suggests that senolytics and antivirotics are potential novel therapeutic avenues.

Glioblastoma (GB; formerly glioblastoma multiforme, GBM)^1^ is the most aggressive and lethal form of human brain tumor, associated with a poor prognosis. Despite surgery resection followed by radiochemotherapy (RCHT), the overall survival ranges between 14 and 16 months.^2^ GB exhibits highly invasive diffuse growth, with tumor cells infiltrating along blood vessels and white matter tracts deep into healthy brain tissue,^3^ rendering complete resection unfeasible. Additionally, despite post-surgery RCHT, GB progresses rapidly and leads to death, demonstrating pronounced resistance to standard-of-care therapy.^2^ The tumor microenvironment (TME) plays a central role^4^ and, beyond stromal and immune cells, a critical component affecting the TME is the secretory activity of primary and secondary, therapy-induced, cancer-associated senescent-like cells. Cellular senescence (CS) promotes the malignant progression of GB via the paracrine effects of senescence-associated secretory phenotype (SASP), including pro-angiogenic proteins and senescence-associated cytokines.^5^ The high presence of senescent cells (SCs) negatively correlated with patients’ survival. Their removal in a mouse GB model increased animal survival.^6^ Additionally, CS is the primary response to ionizing radiation (IR) in GB.^7^ The increase in the number of SCs in GB after irradiation is likely one of the main reasons for tumor recurrence^8^ and their selective elimination can prevent recurrence in mouse GB models.^9^^,^^10^ Temozolomide (TMZ), an alkylating agent used in the chemotherapy of GB, induced senescence in GB cells dependent on functional p53 and sustained expression of p21^waf1/cip1^ (p21).^11-13^ Induction of p21-dependent CS in GB leads to increased tumor growth and migration.^10^ Therefore, GB patients could benefit from senolytic therapy. Compounds exhibiting senolytic activity can induce death of senescent GB cells, including navitoclax, chloroquine, and natural compounds fisetin and artesunate.^14^ Navitoclax treatment selectively killed SCs in a mouse and attenuated the growth of GB cells implanted into pre-irradiated brains.^10^ It is worth noting that a platform for targeted administration of a senolytic agents potentially useful in the treatment of GB was developed, based on the selective accumulation of lipofuscin in SCs.^15^^,^^16^ This approach increases the selectivity of senolytic therapy towards SCs. It thereby reduces the side effects associated with current senolytic interventions. A clinical trial testing senolytics in the GB treatment has recently been initiated (ID: NCT07025226).

Viral involvement in GB pathogenesis has garnered increasing attention. Several viral species have been detected in GB biopsy specimens, suggesting their potential role in tumor development. For instance, polyomaviruses, papillomaviruses, and members of the Herpesviridae family, including human cytomegalovirus (HCMV), Epstein-Barr (EBV), and herpes simplex (HSV) viruses, have been detected in GB.^17^ Significantly, adjuvant treatment with the antivirotics valganciclovir has been associated with prolonged overall survival in GB patients, suggesting a therapeutic benefit of targeting viral components in GB management.^18^

This study aimed to use multi‑omics integration of publicly available datasets from GB patients, with a focus on identifying SCs and their potential role in GB pathogenesis. We defined a core set of “common” CS markers shared across various non-GB SCs types. We applied this marker set, along with co-expression of SASP, extracellular matrix (ECM) components, and cyclin-dependent kinase inhibitors (CDKi), to GB datasets. Using this approach, we identified cell subpopulations within GB tumors exhibiting senescent characteristics and confirmed the presence of SCs in primary human GB biopsies. These findings enabled us to characterize the molecular features of GB-associated SCs and investigate their role in GB development. Furthermore, we identified CS and antiviral response as features distinguishing GB from less aggressive low-grade gliomas. Notably, features indicative of senescence-escaping cells have been identified only in GB. Experimentally, we validated our mRNA-based CS marker set through proteomic analysis of an in vitro TMZ-induced senescent GB cell line. These findings also suggest that chemotherapy may further enhance senescent traits in GB, reinforcing the relevance of CS in GB progression and therapy responses.

## Methods

For a description of cell cultures, senescence induction and determination, preparation of spheroids, mass spectrometry (MS) sample preparation and analysis, and bioinformatic data analysis, see Supplementary Methods.

### Data Processing

To define CS markers, 6 publicly available transcriptomic datasets of senescent and proliferating cells were used. Each dataset contains a different cell type and different types of CS, including replicative CS of HMEC (human mammary epithelial cells, E-GEOD-16058), replicative CS of HCAECS (human coronary artery endothelial cells, E-GEOD-77239), replicative CS of PTEC (proximal tubule epithelial cells, E-MEXP-2683), replicative CS of hMSC (human mesenchymal stem cells, GSE35957), Ras-induced CS of IMR-90 (E-GEOD-19864), and erlotinib-induced CS of NHBE (normal human bronchial epithelial cells, GSE100014) cells. Only young and SCs from datasets E-GEOD-19864 and E-MEXP-2683 were analyzed. Only untreated senescent and young cells were extracted from the E-GEOD-77239 dataset. In total, the datasets contain 2 (E-MEXP-2683, E-GEOD-19864), 3 (E-GEOD-77239, GSE100014), 4 (E-GEOD-16058), and 5 (GSE35957) experimental replicates.

Publicly available single-cell transcriptome of GB cells^19^ contains 32 877 cells across 11 patients’ tumors, with 17 676 genes analyzed.

ECM components were defined based on MatrixDB.^20^ Genes upregulated in IMR-90 10 days post-irradiation (log_2_FC > 0.5), available in the SASP Atlas,^21^ were taken as SASP representatives. We considered the top 15% of cells expressing the most ECM or SASP components as cells with high ECM or SASP expression.

The Ivy GB Atlas^22^ contains transcriptomic profiles of anatomical regions, including *Cellular Tumor*, *Perinecrotic Zone*, and *Leading Edge*, from 41 tumors. To identify regions that most closely match the senescent profile, we scaled the gene intensities of selected genes to 0-1, and the values of downregulated markers were multiplied by −1. Then, we summed up the values for a given set of genes (senescent markers, SASP, ECM components). The region with the highest sum expresses the given set of genes the most.

The publicly available transcriptomic profiles of GBs and low-grade gliomas (GSE4290^23^) contains non-tumor samples (*n* = 23; epilepsy patients), grade II (*n* = 7) and grade III (*n* = 19) astrocytomas, grade II (*n* = 38) and grade III (*n* = 12) oligodendrogliomas, GBs (*n* = 77), and 4 samples without a clear diagnosis, which were removed. Astrocytomas and oligodendrogliomas were grouped by grade into grade II (*n* = 45) and III (*n* = 31) to ensure sufficient statistical power and stable results reflecting robust molecular programs associated with tumor grade rather than specific subtypes in differential expression and downstream metabolic pathway analyses.

The publicly available transcriptome, proteome, and phosphoproteome of 99 GB patient samples and 10 normal brain samples were used to calculate mRNA-protein correlations and identify phosphorylations.^24^

### Statistics

The statistical analyses were performed using Python^25^ (RRID: SCR_008394) and R^26^ (RRID: SCR_001905), for a detailed description of used libraries, see Supplementary Methods. A linear model with empirical Bayes moderation was used for differential gene expression analysis, and the false discovery rate method was used to adjust the *P* value. Differentially expressed genes (DEGs) have adjusted *P* < .05 and an absolute log_2_FC > 0.5. The cut-off for significant pathways from the Kyoto Encyclopedia of Genes and Genomes (KEGG; RRID: SCR_012773) was set at adjusted *P* < .05. Significantly enriched genes with an absolute log_2_FC value greater than 1 were used in the Signaling pathway impact analysis (SPIA). The magnitude of *P*-values is indicated using asterisk (*.01≤*P < *.05; **.001≤*P < *.01; ***.0001≤*P < *.001; ****P < .0001). *P*-values higher than 5e-02 are marked “ns,” which indicates a nonsignificant change.

## Results

### Definition of “Common” Senescence Markers

A critical step in studying the effect of CS on pathophysiology is the reliable identification of SCs in tissues. Due to the absence of specific markers of senescence,^27^ we adopted an approach based on shared transcriptome signatures derived from other types of SCs to identify their presence in GB. To define senescent signatures, 6 transcriptomic datasets comparing SCs with their proliferating counterparts were analyzed (see Methods for the datasets used).

**Figure 1.**
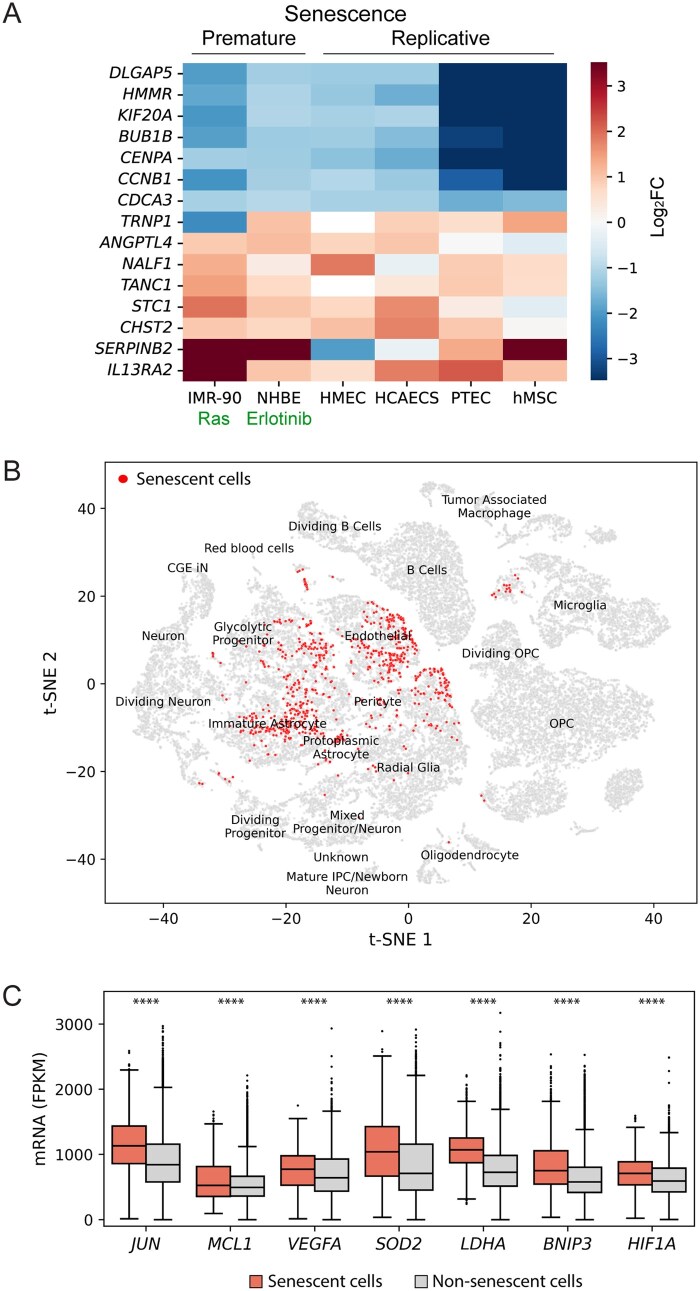
(A) Heatmap of gene expression of “common” senescence markers presented as log_2_FC (color range) across 4 replicative senescence and 2 premature senescence cell models. “Common” senescence markers contain 8 most upregulated based on FC > 1.7 in at least 4 of the 6 datasets and 7 most downregulated showing FC < 0.5 across all 6 datasets. The x-axis shows the cell lines analyzed, and the y-axis shows the of “common” senescence markers. (B) Visualization of cells appears to be GB-associated SCs (836 cells) based on the simultaneous expression of the curated set of “common” senescence markers, SASP, ECM components, and CDKi in the single-cell GB transcriptome. (C) Expression of the anti-apoptotic gene *MCL1*, proto-oncogene *JUN* and genes associated with hypoxia (*HIF1A*, *BNIP3*, *VEGFA*), anaerobic glycolysis (*LDHA*), and oxidative stress (*SOD2*) which are upregulated in GB-associated SCs compared to non-SCs. Statistical significance among groups was analyzed by Mann-Whitney *U* test and significant changes are indicated by asterisks (****P < .0001).

Across all datasets, 10 691 genes were significantly differentially expressed and 589 genes showed consistent deregulation (upregulation or downregulation) across at least 3 datasets. Of these 589 putative senescence markers, 128 genes were consistently upregulated and 461 downregulated in SCs compared to their proliferating counterparts (Supplementary Table S1). To identify SCs in single-cell transcriptomes, we defined a set of “common” senescence markers comprising 8 most upregulated and 7 most downregulated genes (Figure 1A).

### Identification of SCs in GB Single-Cell Transcriptome

Next we defined specific GB-associated SC populations utilizing a single-cell RNA sequencing dataset.^19^ Cells from this dataset form 21 clusters named after characteristic expression profiles, corresponding to the developmental stages of normal brain (Supplementary Figure S1A).

We defined 4 criteria for identifying SCs in the transcriptome. First, the cells’ expression must be consistent with “common” senescence markers. The cumulative expression of the upregulated CS genes exceeding 1000, and simultaneously the absence of the expression of the downregulated senescent markers is required (Supplementary Figure S1B). The second and third criteria include high expression of ECM^20^ and SASP^21^ components, which are characteristic of SCs (Supplementary Figure S1C-E). The fourth criterion was the expression of INK4/KIP CDKi families involved in cell cycle regulation. As experimental data showed that suprathreshold long-term expression of CDKi is sufficient to induce CS,^28-30^ we assume that the CDKi expression in GB identifies CS.

By combining these 4 criteria, we identified 836 GB-associated SCs (2.5% of the total) that belong to the cell clusters: *Endothelial* (30%), *Immature Astrocytes* (27%), *Radial Glia* (27%), *Protoplasmic Astrocyte* (5%), and rare (3% or fewer) other cluster types (Figure 1B). Additionally, GB-associated SCs compared to non-senescent have higher expression of the anti-apoptotic gene *MCL1*, the proto-oncogene *JUN* and genes associated with hypoxia, anaerobic glycolysis, and oxidative stress (Figure 1C).

Notably, a subpopulation (15%) of cells expressing *p21* and SASP also express the commonly used proliferation marker *MKI67* (*Ki67*; Supplementary Figure S2A). These cells belong primarily to *Dividing neuron* (28%) and *Dividing Progenitor* (24%), followed by *Microglia* (15%). Additionally, most of these *Ki67*-expressing SCs co-express the replication factor *PCNA* (75%) and the MCM complex (89%), indicating that these cells proliferate and may have escaped CS.

Altogether, these findings indicate the presence of cells with a characteristic senescence-associated gene expression pattern in GB patient samples. The main SCs subpopulations were *Endothelial*, *Immature Astrocytes*, and *Radial Glia*, based on the transcription profiles of the corresponding cell types in the developing brain.

### SC Communication Network in GB

**Figure 2.**
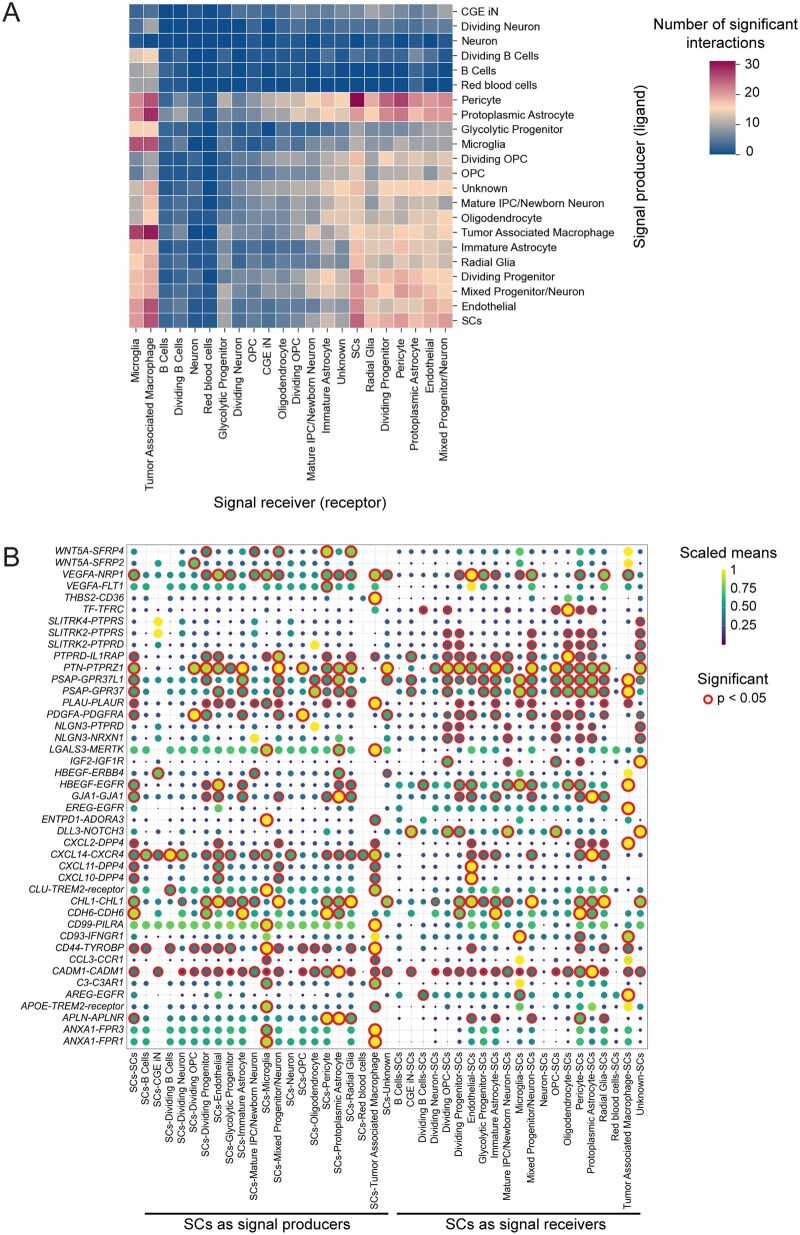
(A) Asymmetric heatmap showing the number of significant interactions between individual clusters. On the x-axis are the signal-receiving clusters (receptor), and on the y-axis are the signaling clusters (ligand). (B) Significant interactions of GB-associated senescent cells (SCs) with other clusters, where each dot represents a ligand-receptor interaction (defined on y-axis) between the sending and receiving clusters (on the x-axis). The color scale of the dots indicates interaction strength, quantified as the scaled mean expression of ligand and receptor genes. The size of the dot reflects a combination of interaction strength (scaled means) and statistical significance (-log_10_*P*), such that larger dots correspond to stronger and more statistically confident interactions. Statistically significant interactions (*P* < .05) are highlighted. Statistical significance was analyzed by permutation test.

SCs can modulate TME through a wide range of factors, including predominantly components of SASP. To investigate the specific interactions between SCs and other cell types in GB, we analyzed the communication of 836 SCs (identified in the single-cell dataset) by identifying ligand-receptor pairs across different cell clusters. As shown in Figure 2A, SCs exert the most robust impact on immune cells, as indicated by the number of significant interactions, with TAM and, to a slightly lesser extent, microglia being most affected, mediated mainly by SASP components, including ANXA1, C3, CXCL2, CXCL14, and LGALS3 (Figure 2B). SCs receive most signals from the *Pericyte* cluster, via, e.g. APLN-APLNR, VEGFA-NRP1, and VEGFA-FLT1 (see summary in Supplementary Table S2).

Altogether, cell-to-cell communication analysis revealed that SCs interact with other GB cells through an extensive network, primarily receiving signals from the Pericyte cluster and modulating TAM and microglia function via SASP components, including chemokines, suggesting a potential role for senescence in modulating the TME and immunity in GB.

### SCs Are Present in GB Hypoxic Areas

**Figure 3.**
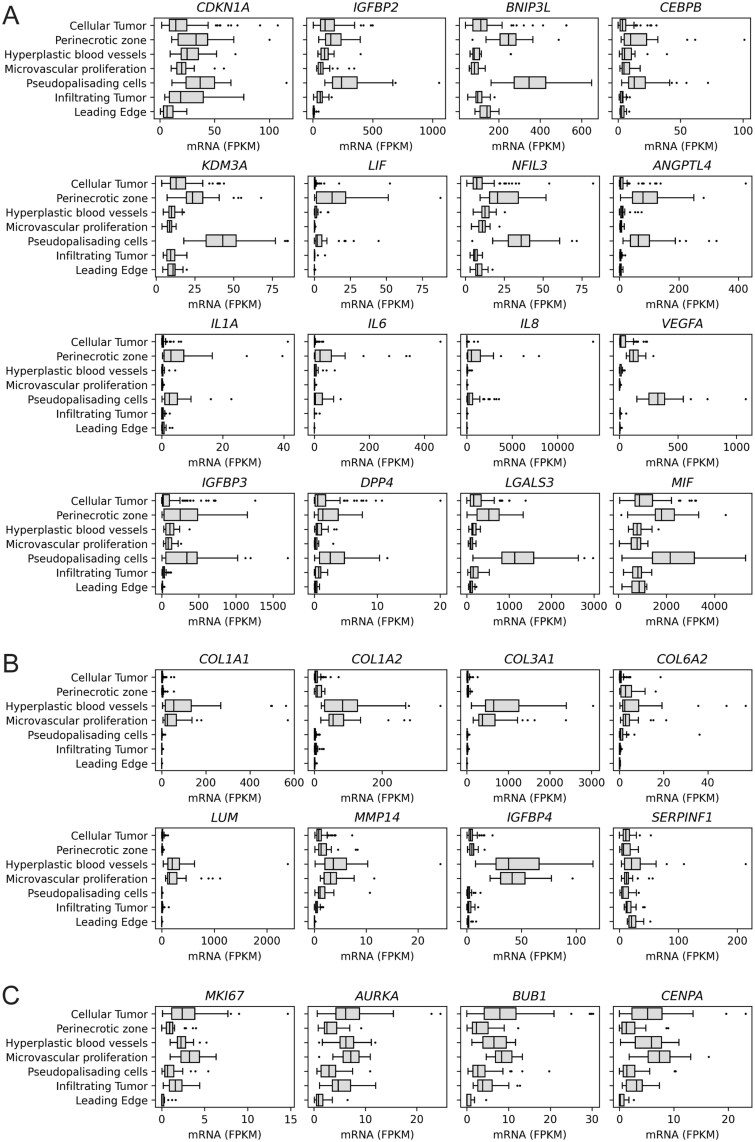
Expression of selected genes in different histologically defined areas of GB. (A) Senescence-associated genes, SASP genes, ECM components, and the proangiogenic factor *VEGFA* that are dominantly expressed in *Pseudopalisading cells* and *Perinecrotic zone*. (B) ECM and SASP components that are dominantly expressed in *Hyperplastic blood vessels*. (C) Expression of proliferatione marker *Ki67* (*MKI67*) and genes involved in cell cycle regulation in different histologically defined areas of GB. (A-C) The x-axis shows gene-specific mRNA expression, quantified as fragments per kilobase of transcript per million mapped reads (FPKM), and the y-axis shows the histologically defined areas.

Next, we analyzed the transcriptomic data^22^ of samples from different histologically defined areas of GB, to assess SCs marker expression. As a marker set, we used all 128 upregulated genes and the 133 most downregulated genes associated with CS, as defined in the first Results section. Here, we found GB regions that most closely match the SCs profile, that is, those that highly express upregulated and lack the expression of downregulated senescence markers. *Leading Edge* cells, followed by the *Perinecrotic zone* and *Pseudopalisading cells*, exhibited the highest senescence score. Note, that the leading edge is comprised of invasive tumor cells infiltrating the surrounding healthy brain tissue (approximately 1-3 tumor cells per 100 healthy non-dividing brain cells). Therefore, the apparently high senescence marker score in the leading edge likely reflects the dominance of nonproliferating brain cells (infiltrated by the much less abundant GB cells) in the absence of proliferation markers typically found in healthy brain tissue, rather than genuine CS. The pseudopalisading cells are hypoxic tumor cells that actively migrate away from the perinecrotic zone and form layers around the necrotic area. Upregulated senescence markers are predominantly expressed in *Pseudopalisading cells* and *Perinecrotic zone*, including *p21* and interleukins. Several SASP and ECM components are also predominantly expressed in these areas, including *LGALS3* and *VEGFA* (Figure 3A). Nevertheless, SASP and ECM components, such as collagens, are mostly expressed in *Hyperplastic blood vessels* (Figure 3B). However, these cells express proliferative marker *Ki67* and genes involved in cell cycle regulation, which are typically downregulated in CS (Figure 3C).

Our findings indicate that most SCs occur in tumor hypoxic areas, in perinecrotic and pseudopalisading cell zones. Moreover, abundant expression of SASP and ECM components is observed in proliferating cells forming hyperplastic blood vessels.

### CS and Antiviral Responses Are Unique for GB

**Figure 4.**
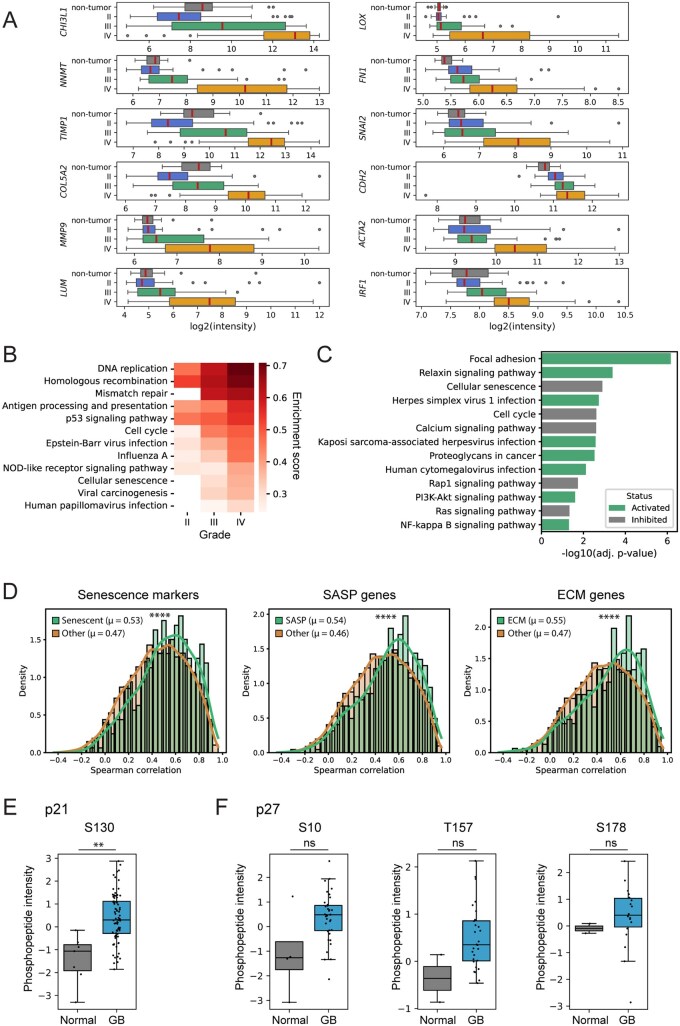
(A) Expression of individual genes in normal brain, low-grade gliomas, and GB. (B) Heatmap with enrichment scores of KEGG pathways in GB and lower-grade gliomas, where the intensity of the pathways increases with increasing malignancy. (A and B) Grade II and III (low-grade gliomas) include astrocytomas (grade II: *n* = 7; grade III: *n* = 19) and oligodendrogliomas (grade II: *n* = 38; grade III: *n* = 12). Grade IV includes only GBs (*n* = 77). (C) Examples of uniquely activated and inhibited KEGG signaling pathways in GB according to SPIA. (D) Distribution of Spearman correlation coefficients for mRNA-protein correlations of individual genes in individual sets of 589 senescence markers, SASP, and ECM components, and the remaining genes in the dataset. The median of the distribution is specified in parentheses. (E and F) Normalized phosphopeptide log2 intensity of p21 (CDKN1A) phosphorylated at serine 130 (E) and p27 (CDKN1B) phosphorylated at serine 10, threonine 157, and serine 178 (F) in normal brain and GB. (D and F) Significantly different distributions and significant changes are indicated by asterisks (**P < .01, ****P < .0001), and “ns” indicates a nonsignificant change. Mann-Whitney U test (D) and Welch’s *t*-test (E-F) were used for statistics.

To identify processes that differentiate GB from lower-grade gliomas, we analyzed a transcriptome of non-tumor brain samples, grade II-III gliomas, and GBs.^23^ Among the 4,493 DEGs in GB compared with nontumor samples, 73% are shared with DEGs found in lower-grade gliomas (Supplementary Figure S3A and B). Thus, 1207 genes were uniquely enriched in GB, of which the most upregulated are *CHI3L1*, *NNMT*, ECM remodeling genes, collagens, matrix metalloproteinases, the key epithelial-mesenchymal transition (EMT) transcription factor *SNAI2* and genes associated with mesenchymal phenotype (Figure 4A). Another difference between GB and low-grade gliomas was the GB-associated upregulation of *IRF1*, which plays a key role in the activation of antiviral genes (Figure 4A).

Based on gene set enrichment analysis (GSEA), multiple pathways that are upregulated in II-IV gliomas compared to non-tumor samples show increasing intensity with increasing malignancy (Figure 4B). These pathways are consistent with the higher aggressiveness of IV-grade tumors. The levels of some pathways increase to such an extent that they are significantly higher in GB than in lower-grade gliomas, including the PI3K-Akt, HIF-1, Toll-like receptor, JAK-STAT, and MAPK signaling pathways. The most upregulated signaling pathways in GB compared to II-III grade gliomas include those associated with antiviral response and CS, such as *Cellular senescence*, *Viral carcinogenesis*, the *p53 signaling pathway* and pathways for EBV, HSV, Kaposi sarcoma-associated herpesvirus (KSHV), and papillomavirus infection. Furthermore, 26 pathways are significantly enriched only in GB (Supplementary Figure S3C), including *Human cytomegalovirus infection*.

SPIA performed to characterize further the role of these deregulated pathways in GB pathogenesis, identified 34 pathways that were significantly activated and 40 that were significantly inhibited in GB. The activation of 28 and the inhibition of 21 signaling pathways are unique to GB (Figure 4C). Uniquely activated include signaling pathways for HSV, KSHV, and HCMV infection, and uniquely inhibited include the *Cellular senescence*. The paradoxical inclusion of CS among the inhibited processes by SPIA is due to the activated the *PI3K-Akt signaling pathway*, which can inhibit senescence^31^ via inhibitory phosphorylation of p21,^32^ p27,^33^ and FOXO3.^31^

Based on the transcriptomics differences between GB and lower-grade gliomas, GB exhibits a unique, high level of senescence markers. Furthermore, GB is unique in robustly activating the antiviral response, EMT, extensive ECM remodeling, and escape from CS.

### Correlation between mRNA and Protein Levels of Senescence Markers

Using a dataset containing transcriptome and proteome data for each GB patient,^24^ we assessed the correlation between mRNA and protein levels of all 589 putative senescence markers (Supplementary Table S1), SASP, and ECM genes. All exhibit significantly higher Spearman correlations between transcript and protein levels compared to other genes (Figure 4D). Overall, 88.3% of senescence markers, 87.3% of SASP genes, and 86.9% of ECM genes with available mRNA and protein level data showed a significant Spearman correlation (*P* < .05), indicating strong concordance between transcript and protein levels. Therefore, the transcriptome data used in the earlier analyses of our present study are reliable for detecting SCs.

### Inhibitory Phosphorylation of p21 and p27 in GB Specimens

The presence of cells dual-positive for *p21* and the *Ki67*, along with a concomitant SASP transcriptomic signature in the single-cell data, suggests that these cells have escaped CS. Among potential mechanisms responsible for the escape is the inactivation of CDKi through subcellular sequestration or degradation. Therefore, we leveraged a phosphoproteomic dataset from GB samples^24^ and analyzed inhibitory CDKi phosphorylation. Indeed, p21 was phosphorylated at serine 130 in 82% of GB samples, and this phosphorylation was significantly higher compared to normal brain (Figure 4E). Moreover, p27 was phosphorylated at serine 10, threonine 157, and serine 178 (Figure 4F) in 36%, 28%, and 18% of GB samples, respectively.

### Chemotherapy-induced and Primary Senescence in GB Overlap Phenotypically

Because CS can develop in response to RCHT, we further analyzed differences between primary and therapy-induced CS in GB. Given that patient samples are difficult to obtain immediately after RCHT, we induced CS in the U87 GB cells in vitro with TMZ.^34^ We prepared spheroids from them (see Supplementary Methods and Supplementary Figure S4A-J) and, after an additional 7 days, subjected them to MS-based proteomics analysis. The advantage of a 3D approach over 2D cell cultures, in addition to a less artificial environment, was that the secretome remained trapped within the spheroid, eliminating the need to isolate and analyze it separately.

**Figure 5.**
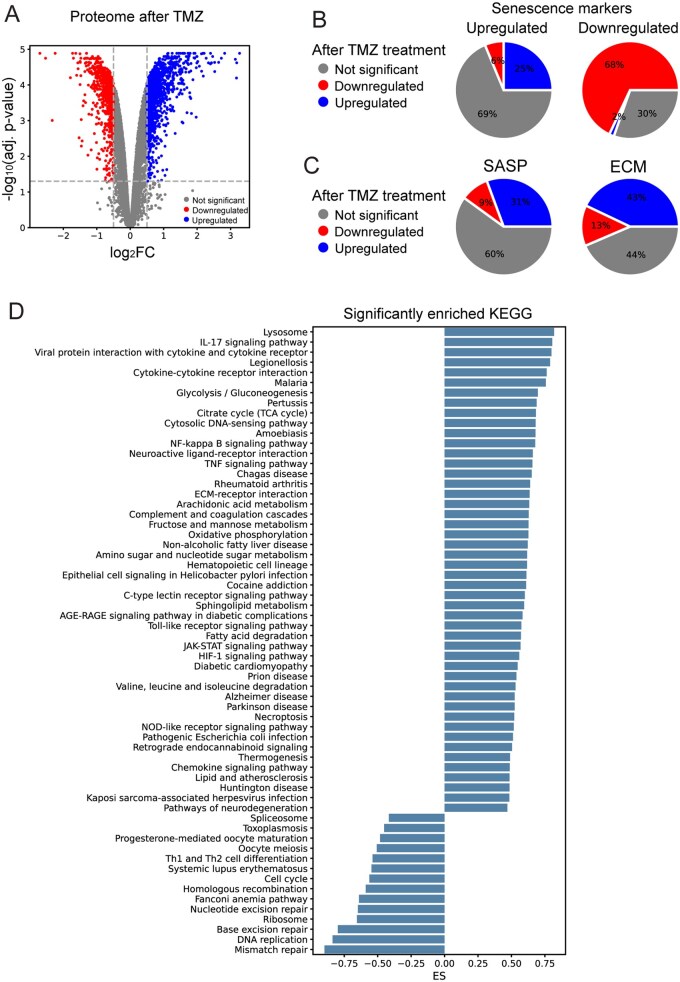
(A) Volcano plot comparing the proteome of 3 biological replicates of 3D U87 spheroids before and after TMZ treatment. (B and C) Pie charts showing how downregulated and upregulated senescence markers (B), SASP, and ECM components (C) behave in the proteome after TMZ treatment. (A-C) Significantly downregulated genes after TMZ treatment are highlighted in red, upregulated genes in blue, and genes without significant change in gray. (D) Enrichment scores (ES) of significantly enriched KEGG signaling pathways after TMZ treatment.

We identified 1188 significantly upregulated and 748 significantly downregulated proteins in TMZ-treated U87 spheroids compared to untreated controls (Figure 5A). The most upregulated were cytokines and chemokines, such as CSF3, IL1B, CXCL8, and IL6. The downregulated proteins are primarily involved in cell cycle regulation, including STMN1, PCLAF, and SPC24. Most senescence markers (53% of all 589 senescence markers in Supplementary Table S1), defined by transcriptomics datasets, are significantly deregulated after TMZ, which supports the development of a senescent phenotype. Specifically, 25% of upregulated senescence markers are upregulated and 68% of downregulated senescence markers are downregulated (Figure 5B). Therefore, TMZ-treated U87 SCs exhibit senescence markers shared with different senescence models. Moreover, many SASP and ECM proteins were upregulated (Figure 5C), again consistent with the development of a senescent phenotype.

Furthermore, to identify processes affected by the TMZ, we performed a GSEA. Upregulated KEGG pathways are associated with senescence and antiviral/pathogen response (Figure 5D). Intriguingly, several of these pathways distinguish GB from lower-grade gliomas, suggesting they are pivotal for GB aggressiveness. Downregulated pathways are associated with cell proliferation, which underscores the development of CS in TMZ-treated GB cells.

In conclusion, TMZ induces a senescent phenotype in a human GB line in vitro, characterized by the expression of senescence markers commonly observed in other types of senescence, as well as the upregulation of SASP and ECM components. These findings also validate the criteria used to identify GB-associated SCs, indicating that primary and secondary therapy-induced senescence in GB shares similar phenotypic features. Moreover, several signaling pathways unique to GB aggressiveness are upregulated after TMZ treatment.

## Discussion

CS plays multiple and often conflicting roles in the complex biology of solid tumors, from inhibiting proliferation in benign stages to supporting malignant features in more advanced tumors. Moreover, SCs influence the TME with rich secretome. CS can occur in both normal and tumor cells, either endogenously or secondarily in response to exogenous stress factors, primarily RCHT. In normal cells, the senescent state is semi-stable, with a low probability of spontaneous escape, whereas in tumor cells, the senescent phenotype is unstable and therefore characterized as senescent-like.^35^^,^^36^

In this study, we aimed to determine, using multi‑omics integration, whether SCs are present in GB before RCHT treatment, to estimate their impact on GB pathogenesis, and to experimentally evaluate the effect of chemotherapy in vitro on human GB cells from the perspective of the development of secondary therapy-related senescence.

### Identification of SCs in GB Transcriptome Datasets

Using relatively stringent criteria, we have identified several markers whose deregulation is shared across different cell types developing CS in vitro via various mechanisms. We used the deregulated senescence markers combined with 3 other criteria of enhanced expression of characteristic senescent traits, comprising SASP, ECM components, and the expression of at least one CDKi, to identify GB-associated senescent cells in the GB single-cell transcriptome. The highest proportion of SCs was found in the *Endothelial* cluster, followed by *Immature Astrocytes* and *Radial Glia*. These cells co-express, besides genes associated with hypoxia, anaerobic glycolysis, and oxidative stress, the antiapoptotic factor *MCL1,* suggesting their resistance to cell death may be circumvented with MCL-1 inhibitors. Moreover, Borovski et al. demonstrated that endothelial cells are highly resistant to IR, undergoing CS rather than cell death. The endothelial SCs support the expansion of GB stem cells,^37^ suggesting communication between these 2 GB cellular compartments. Recently, the SenPy tool was published,^38^ focusing on identifying SCs based on transcriptomic signatures in single-cell transcriptomic data. This algorithm aligns with our strategy and independently supports the robustness of our approach. Moreover, it highlights the community’s interest in transcriptomic definitions of CS, as the use of known markers, such as p16, proves insufficient.

Our analysis of GB-associated SCs interactions with other tumor cells indicates that SCs exhibit rather tumor-promoting effects. For instance, SCs affect nearly all cells, including themselves, through CXCL14-CXCR4 interaction. CXCL14 chemokine signaling is associated with cell invasion, growth, recurrence, and poor survival in GB.^39^ SCs express DPP4, which post-translationally modifies chemokines essential for recruiting immune cells into the TME, CXCL10 and CXCL11, produced by senescent and endothelial cells. DPP4-mediated modification of these chemokines reduces immune cell infiltration.^40^ DPP4 inhibition improves antitumor responses in mouse models of hepatocellular carcinoma and breast cancer.^41^ High DPP4 expression is associated with high pathological glioma grade and with poor prognosis in low-grade gliomas.^42^ Another example is PLAU-PLAUR interaction, primarily affecting TAM and microglia, but also the endothelium, activating inflammatory processes and tumor-related signaling pathways.^43^ The PLAU-PLAUR interaction-mediated signaling is associated with poor prognosis in primary and recurrent gliomas, and PLAU and PLAUR expression is increased in higher-grade primary gliomas and in recurrent gliomas.^41^ Last but not least, SCs can stimulate endothelial cell migration and angiogenesis via VEGFA-NRP1 interaction.^44^ Altogether, based on our analysis of cell communications, SCs negatively modulate the TME in GB mainly through SASP, thereby promoting immune suppression, tumor growth, aggressiveness, angiogenesis, and chemoresistance (Supplementary Table S2).

Transcriptome analysis of different histological regions of GB revealed that the most pronounced senescent populations appear to be hypoxic cells from the perinecrotic zone and hypoxic cells that actively migrate away from the perinecrotic zone, pseudopalisading cells. These cells express *p21*, senescence markers, interleukins, SASP, and ECM components. GB is characterized by hypoxic areas, a feature that contributes to GB aggressiveness, as hypoxic areas produce a resistant tumor cell population^45^ and increase the expression of EMT genes, leading to cells acquiring stemness properties.^46^ Notably, some herpesviruses, such as KSHV, HSV, EBV, and HCMV, can be reactivated under hypoxic conditions, and hypoxia modulates the microenvironment to support viral replication.^47^ Additionally, pseudopalisading cells abundantly express *VEGFA*, which induces endothelial proliferation and promotes the formation of new hyperplastic blood vessels, suggesting that this senescent area may drive vascular remodeling.

In a dataset by Wang et al. we found a strong correlation between transcript and protein levels for senescence markers, SASP, and ECM components, indicating that transcriptome data are a reliable source for detecting SCs.

### CS and Antiviral Responses Are Unique for GB

Transcriptome analysis of low-grade gliomas and GB allowed us to identify GB-specific processes. Active signaling pathways that distinguish GB from lower-grade gliomas generally signal a response to herpesvirus infection, such as those triggered by HSV, KSHV, and HCMV, as well as other pathways related to viral infection, including *Focal adhesion*, the *NF-kappa B signaling* and the *PI3K-Akt signaling pathways*. Other virus-related pathways are significantly activated in grade III-IV gliomas, with greater robustness in GB, including pathways for papillomavirus, EBV, *Antigen processing and presentation,* and *MAPK signaling pathway*. For instance, ERK-MAPK activation drives HCMV reactivation,^48^ and depending on this activation, HCMV protects infected myeloid cells from induced cell death by upregulating MCL-1.^49^ We observed enhanced expression of *MCL1* in GB-associated SCs. Note that activation of virus-specific pathways (as KEGG pathways include, besides general antiviral responses, also virus-specific signatures) suggests coinfection of GB with herpesviruses. This finding is consistent with the identification of herpesviruses in GB patient samples and the fact that almost the entire adult human population is infected with one or more herpesviruses.^17^

Moreover, the *Cell cycle* appears to be inhibited in GB, as evidenced by the upregulation of *p53* and CDKi (*p21*, *p16*, and *p18*), key factors in the development of CS. However, SPIA indicates inhibition of CS despite some key senescence drivers being upregulated, including *p53* and *p21*, as supported by markers of ongoing cell cycle, including CDKs, cyclins, MCMs, *PCNA*, and *MKI67*. The senescence suppression could be mediated by the activity of the PI3K-Akt signaling pathway, which can lead to the escape of SCs from senescence.^31^^,^^50^ Besides the frequent (up to 32% of primary GB^51^) activation of the PI3K-Akt by mutation or depletion of PTEN,^52^ the PI3K-Akt can be activated during the infection with herpesviruses, for instance, by HCMV infection.^53^

Interferon regulatory factors (IRFs) are key components in defense against viral infection by activating IFN responses.^54^  *IRF1* is a gene expressed as part of the late IFN-gamma response and a potent cell proliferation suppressor,^55^ whilst *IRF3* activates IFN-alpha and IFN-beta responses.^54^ Upregulation of *IRF1* and *IRF3* is unique to GB compared to lower-grade gliomas, suggesting active viral infection. We found that *IRF1* is also overexpressed in hypoxic-senescent areas, in pseudopalisading cells and perinecrotic zone. In the single-cell transcriptome, 58% of identified SCs express *IRF1*. To overcome IRF1-mediated antiviral responses, some virus species, have evolved mechanisms to suppress the IRF1-mediated interferon signaling. For instance, KSHV inhibits the IFN signaling using the viral vIRF.^56^ Additionally, in the Wang et al. dataset, we identified phosphorylation of IRF3 at serine 175. The HSV US3 kinase is the only known kinase that mediates this phosphorylation, thereby preventing IRF3 activation and IFN-β production.^57^ These findings explain why we did not identify the downstream activation of the interferon signaling pathway in any GB dataset.

The gradually increasing intensity of key signaling pathways associated with GB aggressiveness (Figure 4B) suggests a gradual progression of some lower-grade gliomas into GB, rather than 2 independent tumor evolutionary paths. If this were the case, then senolytic and antiviral treatment could be beneficial even for patients with lower-grade gliomas to prevent progression towards secondary GB.

### Putative Mechanisms of Senescence Escape in GB and the Role of Viral Infection

Only a small fraction (6%) of p21-positive cells in the single-cell transcriptome exhibited a “complete” senescent phenotype. Notably, some *p21*-positive cells exhibit a mixed phenotype, characterized by the co-expression of proliferation and senescence markers, indicating an escape from CS. For instance, a subpopulation of dividing neurons and progenitor cells expresses *p21*, *MKI67*, *PCNA*, MCMs, SASP components, and mesenchymal genes. Moreover, proliferating cells forming hyperplastic blood vessels around hypoxic senescent areas exhibit high SASP expression, suggesting that they are candidates for post-SCs. SCs that escape cell-cycle arrest promote chemoresistance and highly aggressive growth in recurrent tumors, owing to their stem-like phenotype.^58^

One possible mechanism of senescence escape is the inactivation of CDKi, including p21 and p27. Supporting this idea, 82% of GB patients from the analyzed dataset^24^ carry p21 serine 130 inhibitory phosphorylation. Under normal cell cycle progression, this phosphorylation mediated by the CDK1/cyclin B complex leads to p21 degradation, enabling the cell to enter mitosis. However, we did not detect any CDK1-activating phosphorylation in patients’ samples, nor in other cell cycle-related CDKs. In the context of viral infections, HCMV induces cyclin E expression and activates the CDK2/E complex,^59^ which phosphorylates p21 on serine 130.^60^ However, this possibility is less likely given the absence of CDK2-activating phosphorylation and presence of CDK2-inhibitory phosphorylation in the GB samples from the dataset of Wang et al. though it cannot be ruled out. Nevertheless, HCMV gene products can activate kinase ERK2,^61^ leading to the phosphorylation of p21 on serine 130.^62^ In 82% of GB patients, we found activating dual phosphorylation of ERK2 on threonine 185 and tyrosine 187. However, whether this p21-inhibitory phosphorylation is related to HCMV infection in GB remains to be verified. It should be emphasized that p21 serine 130 phosphorylation is known to be induced by herpesviruses to help them overcome the p21-dependent cell cycle arrest.^63^ Moreover, we detected p27 serine 10 phosphorylation, a modification associated with the suppression of p27 antiproliferative functions during latent KSHV infection.^64^ Note that ERK2 mediates the detected p27 inhibitory phosphorylation at serine 178, analogous to p21 serine 130 phosphorylation.^65^ In addition to p21 and p27 inactivation, other virus-related mechanisms should be considered. For instance, HCMV-mediated activation of the PI3K-Akt signaling during infection^53^ can lead to escape from cell cycle arrest.^31^^,^^50^ In this context, p27 phosphorylation at threonine 157 was observed, which can be mediated by Akt to prevent p27 nuclear localization, thereby suppressing its antiproliferative function.^33^

Moreover, a recent study reports that structural reorganization of the 3D genome architecture is sufficient to drive escape from oncogene-induced senescence.^66^ Therefore, the driving force for senescence escape in GB can also be GB’s extreme genomic plasticity and epigenetic dynamics.

### Chemotherapy-induced and Primary Senescence in GB Overlap Phenotypically

Proteomic analysis of spheroids derived from the TMZ-treated human GB U87 cells revealed that the senescence markers extracted from other senescence types, as well as the SASP and ECM components are deregulated after TMZ which corresponds to a senescent phenotype. The most upregulated immunoregulatory and inflammatory cytokine is IL1B, which promotes glioma cell migration, invasion, and proliferation,^67^ and CXCL8 which promotes survival and migration by inducing EMT in GB cells.^68^ Mesenchymal markers, such as FN1, ACTA2, CDH2, ITGAV, and ITGB5, associated with a stem-like phenotype, are also upregulated. This notion aligns with the phenomenon described as senescence-associated stemness.^58^ The stem-like phenotype and SASP are retained even after overcoming cell cycle arrest, a phenomenon known to be caused by herpesvirus reactivation. Consequently, these post-SCs have the potential to fuel disease relapse, leading to a more aggressive tumor growth.^58^ The most upregulated cytokine pathway after TMZ was the IL-17 signaling pathway which enhances the proliferation and migration of GB cells via the NF-kappa B signaling,^69^ which we also found to be upregulated. The NF-kappa B induces the expression of antiapoptotic genes, such as the BCL2 family,^70^ and therefore is associated with resistance to therapy.^71^^,^^72^ After TMZ, we observed upregulation of BCL2, BCL2L1, and BCL2L2.

Notably, ANGPTL4, identified by us from transcriptomic data analysis as a general senescence marker and upregulated also in proteome after TMZ, is associated with resistance of GB cells.^73^ Importantly, ANGPTL4 has recently been described as a strong pro-inflammatory marker in many different types of SCs, and its knockdown inhibits other pro-inflammatory components of SASP.^74^

It is possible that secondary senescence induced by RT, which was not examined in this study, differs from senescence induced by TMZ. However, therapeutic irradiation of GB cells has been reported to induce a senescence-like phenotype that closely resembles TMZ-induced senescence, including the acquisition of mesenchymal features, activation of SASP, and ECM remodeling, albeit without stable cell cycle arrest.^75^ Notably, this phenotype is associated with increased expression of the cell-surface glycoprotein F3, which is also significantly upregulated by TMZ.

Our findings suggest that primary and secondary therapy-induced senescence in GB shares similar phenotypic features, and if therapy-induced SCs escape a cell cycle arrest, for example, due to viral reactivation, they are candidates for recurrent tumor formation, carrying resistance to subsequent therapy and the ability to modulate immune response through SASP. Importantly, signaling pathways that distinguish GB from low-grade gliomas and contribute to GB’s aggressive phenotype are like those involved in GB’s response to TMZ treatment, suggesting they may play a role in both the unique aggressiveness of GB and the more aggressive behavior of the recurrent tumor.

### Predicting the Role of GB-associated Senescence in GB Pathogenesis

**Figure 6.**
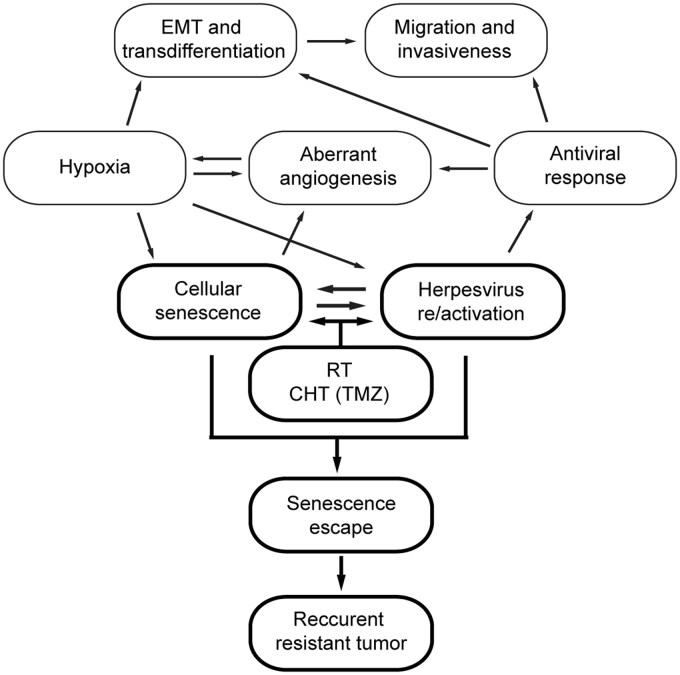
Proposed roles of CS and herpesvirus re/infection among the pathogenic events that occur during GB progression.

One difference between lower-grade gliomas and GB is the higher presence of senescence markers and antiviral responses, suggesting a complex impact of these features on GB malignancy. In Figure 6, we summarize the potential roles of CS and herpesvirus re/infection among the pathogenic events specifically observed in GB.

Determining upstream events that induce CS in clinical settings is challenging, as diverse stimuli can promote CS. Viral pathogens may expand the spectrum of senescence inducers, as exemplified by HCMV infection,^76^ which is involved in GB pathogenesis through proinflammatory cytokines of the antiviral response and the resulting oxidative stress.^77-80^ HCMV has recently been extensively studied in connection with GB pathogenesis and as a novel therapeutic target for GB patients.^81^ It has been verified that HCMV infection promotes angiogenesis,^82^^,^^83^ cell stemness,^84-87^ EMT, and enhances the invasiveness of glioma cells.^88^ Due to the high prevalence of HCMV in the human population, it is unclear how HCMV is reactivated in GB. Mutual link between CS and HCMV reactivation in GB may be senescence-mediated inflammation by pro-inflammatory cytokine secretion or ROS production.^89^ Whether the HCMV reactivation observed during RCHT^90^occurs directly, with or without the contribution of SCs, remains unclear. Nevertheless, HCMV reactivation after RCHT in brain cancer patients leads to cognitively damaging but treatable encephalopathy and premature death.^91^ Additionally, hypoxia, typically associated with GB, may contribute to HCMV re-infection/reactivation, as it modulates the TME that supports viral replication^47^ and can also induce CS.^92^ This idea is consistent with the detection of SCs in hypoxic tumor areas. Finally, CS in GB may be induced secondarily by RCHT, as documented in previous in vitro studies^37^^,^^93^ and supported by our present findings.

Altogether, our bioinformatic analyses of GB patient samples indicate the presence of cells with a senescent phenotype in subpopulations of radial glia, immature astrocytes, and endothelial cells, localized primarily in the hypoxic perinecrotic and pseudopalisading cell zones. These GB-associated SCs negatively modulate the TME in the GB via SASP, thereby promoting immunosuppression, tumor growth, aggressiveness, angiogenesis and chemoresistance, as shown by cell communication analysis. Comparative transcriptome analysis of low-grade gliomas with GB revealed that CS, along with the antiviral response, is the primary feature distinguishing GB from the less malignant gliomas. As suggested, the situation is further complicated because SCs can escape cell cycle arrest and return to the proliferative pool of tumor cells. A notable difference between GB and less malignant gliomas is the presence of senescence-escaping cells, which can be mediated by the inhibitory phosphorylation of p21 and p27 or by the activation of PI3K signaling. These mechanisms belong to the typical host-cell manipulation arsenal of herpesviruses. Moreover, IRF3 phosphorylation identified in several patient samples has, to date, been associated only with the US3 protein of alpha herpesviruses. Our data suggest activation of multiple herpesviruses in GB, such as HCMV, EBV, KSHV, HSV, and the contribution of papillomaviruses cannot be ruled out. RCHT also likely contributes to CS in GB, thereby exacerbating the emerging adverse effects of GB-associated CS on patient prognosis.

## Supplementary Material

vdag122_Supplementary_Data

## Data Availability

Publicly available transcriptome databases analyzed in this study were downloaded from Gene Expression Omnibus (RRID: SCR_005012) and ArrayExpress (RRID: SCR_002964) databases. Their IDs are E-GEOD-16058, E-GEOD-77239, E-MEXP-2683, GSE35957, E-GEOD-19864, and GSE100014. Publicly available single-cell transcriptomics data^19^ analyzed in this study can be browsed at https://cells.ucsc.edu/?ds=gbm. The publicly available proteome-transcriptome data used in this publication were generated by the Clinical Proteomic Tumor Analysis Consortium (CPTAC; RRID: SCR_017135) and are available on the CPTAC Data Portal at: https://cptac-data-portal.georgetown.edu/cptac/s/S048 and Genomic Data Commons (GDC; RRID: SCR_014514) at: https://portal.gdc.cancer.gov/projects/CPTAC-3. A list of extracellular matrix genes used is available in the MatrixDB (RRID: SCR_001727^20^) at http://matrixdb.univ-lyon1.fr/. A list of SASP genes of primary human fibroblasts (IMR-90), 10 days post-irradiation, is available in the SASP Atlas^21^ at http://saspatlas.herokuapp.com/. Ivy GB Atlas^22^ can be browsed at https://glioblastoma.alleninstitute.org/. Transcriptomic dataset of GBs and low-grade gliomas is available on Gene Expression Omnibus (RRID: SCR_005012) with ID GSE4290.^23^ The MS proteomics data have been deposited to the ProteomeXchange Consortium (RRID: SCR_004055; http://proteomecentral.proteomexchange.org) via the PRIDE (RRID: SCR_003411^94^) partner repository with the dataset identifier PXD071225. The experimental metadata has been generated using lesSDRF.^95^
