# Supplementary material for: Cellular senescence escape and antiviral response discriminate glioblastoma from lower-grade gliomas
Source: Neurooncol Adv. 2026 Apr 8;8(1):vdag122. doi: 10.1093/noajnl/vdag122 (PMC13220956; doi:10.1093/noajnl/vdag122)
Supplement: vdag122_Supplementary_Data [file vdag122_supplementary_data.zip › Supplementary_Data (2)/Maurencova_Supplementary_Data_R1_clean.docx]

Supplemental document to original research article

# Cellular senescence escape and antiviral response discriminate glioblastoma from lower-grade gliomas

Dominika Maurencova^1^, Michaela Sadibolova^2^, Monika Zarska^1^, Zuzana Liblova^1^, Josef Novak^1^, Jirina Kroupova^1^, Pavla Vasicova^1^, Jiri Bartek^1,3,4*^, and Zdenek Hodny^1*^

^1^Laboratory of Genome Integrity, Institute of Molecular Genetics of the CAS, Prague, Czech Republic

^2^Biomedical Research Centre, University Hospital Hradec Kralove, Hradec Kralove, Czech Republic

^3^Genome Integrity Unit, Danish Cancer Society Research Center, Copenhagen, Denmark

^4^Division of Genome Biology, Department of Medical Biochemistry and Biophysics, Science of Life Laboratory, Karolinska Institutet, Stockholm, Sweden

**^*^**Correspondence to: Zdenek Hodny, MD, PhD; Laboratory of Genome Integrity,

Institute of Molecular Genetics of the CAS, Videnska 1083, CZ 142 20 Prague 4,

Czech Republic; Tel: (420)-24106 3151, Fax: (420)-24106 2289; E-mail: [hodny@img.cas.cz](mailto:hodny@biomed.cas.cz) and Jiri Bartek, MD, PhD; E-mail: [jb@cancer.dk](mailto:jb@cancer.dk)

# Supplementary Methods

***Cell culture***

A human [GB](https://www.sciencedirect.com/topics/pharmacology-toxicology-and-pharmaceutical-science/colorectal-carcinoma) U-87 MG (U87) cell line (RRID:CVCL_0022, ATCC HTB-14) was obtained from the American Type Culture Collection (ATCC, Manassas, VA, USA). Cells were cultured in the complete growth medium composed of high glucose (4.5 g/L) Dulbecco’s modified Eagle’s medium (DMEM, Gibco/Thermo Fisher Scientific, Waltham, MA, USA), 10% fetal bovine serum (FBS, Gibco/Thermo Fisher Scientific), 100 U/mL penicillin and 100 µg/mL streptomycin sulfate (Gibco/Thermo Fisher Scientific) and 584 mg/L of L-glutamine in the form of L-alanyl-L-glutamine dipeptide (GlutaMAX Supplement, Gibco/Thermo Fisher Scientific). Cells were cultured under a controlled environment (37°C, 5% CO_2_, 95% relative humidity) and were replated at 70–80% confluency by trypsinization. U87 cells were authenticated 6 months prior to the experiment by GENERI BIOTECH (Hradec Králové, Czech Republic). Cells were regularly tested for mycoplasma infection. For biological replicates 1 and 2, U87 cells were defrosted 1 week before use; for biological replicate 3, 8 weeks before use.

***Induction of cellular senescence***

U87 senescent-like cells were prepared by repeated exposure of U87 cells to TMZ (USP, Rockville, MD, USA), diluted in dimethyl sulfoxide (DMSO, Sigma-Aldrich, St. Louis, MO, USA). U87 cells were seeded at 15,000 cells/cm^2^ onto a tissue culture dish (TPP; Trasadingen, Switzerland). After 24 hours, the first dose of TMZ (f.c. 100 µM) was added to cells and then reintroduced during fresh medium replacement every third day (4, 7, and 10 days). For spheroid preparation, senescent-like cells were harvested on day 14 after the first TMZ dose. The development of a senescent-like phenotype was followed by microscopic observation of cell morphology and by determination of senescence-associated β-galactosidase (SA-β-gal) activity and DNA replication using a 5-ethynyl-2’-deoxyuridine (EdU) incorporation assay in all three biological replicates. The coverslips for microscopic analysis were added to the cultivation plastic just before seeding of U87 for TMZ treatment.

***Determination of SA-β-gal activity***

Cellular senescence was confirmed by measuring SA-β-gal activity^1^. Before harvesting the cells used to prepare spheroids, cells growing on coverslips were rinsed with PBS (RT) and fixed with 0.5% glutaraldehyde at RT for 15 min. Cells were washed twice with 1 mM MgCl_2_/PBS, pH 5.8, and incubated in X-Gal staining solution (Fermentas/Thermo Fisher Scientific) at 37°C until the blue color developed. The staining was terminated with three washes in PBS and one rinse in H_2_O. Finally, the cells were mounted with Mowiol (Sigma- Aldrich/Merck) containing 4',6-diamidino-2-phenylindole (DAPI) and imaged on the Leica DM6000 fluorescent microscope using the HC PLAN APO 20×/0.70 DRY PH2 objective and color CCD camera Leica DFC490 (Leica Microsystems GmbH, Wetzlar, Germany). Images were processed with Fiji/ImageJ^2^ (RRID:SCR_002285). To estimate the percentage of SA-β-gal-positive cells, at least 80 cells in each biological replicate were counted.

***Detection of DNA replication by 5-ethynyl-2’-deoxyuridine incorporation assay***

Before harvesting (day 14), the cells used for the spheroids (growing on coverslips) were transferred to DMEM containing 10 μM EdU and incubated for an additional 24 hours. Then cells were rinsed with PBS and fixed with 4% formaldehyde (VWR/Avantor) at room temperature for 15 min. To detect EdU incorporation, click chemistry was performed with Click-iT EdU Cell Proliferation Kit with Alexa Fluor™ 647 dye (Thermo Fisher Scientific) according to the manufacturer’s instructions. The nuclei were stained with 1 µg/mL DAPI (Sigma) in H2O at RT for 3 minutes, then allowed to dry. Finally, the coverslips were mounted in Prolong Gold Antifade mountant (Thermo Fisher Scientific). The stained cells were acquired by high-content imaging using an inverted wide-field microscope (Olympus IX81) equipped with a UPLXAPO 20×/0.8 DRY CORR; FWD 0.6; CG 0–2 objective and a sCMOS camera Hamamatsu ORCA-Fusion BT, 6.5 µm pixel, QE >95%, cooled to -8°C, resolution 2304 x 2304 pixels. The data were analyzed using ScanR Analysis software (Olympus Corporation, Tokyo, Japan). Images were processed using Fiji/ImageJ.

***Preparation of spheroids***

Spheroids were prepared on 96-well plates coated with a thin layer of poly(2-hydroxyethyl methacrylate (poly-HEMA). To prepare the plates, 60 µL of a poly-HEMA solution (5 mg/mL in 96% ethanol) was applied on the bottom of each well of a 96-well round bottom plate. The well plates with lids were placed in a laminar flow box to completely evaporate ethanol, then stored at 4°C. Immediately before use, plates were irradiated at 254 nm for 30 min. To form spheroids, 1,000 U87 proliferating cells (harvested two days after seeding) or 10,000 U87 TMZ-induced senescent-like cells were seeded in 200 µL of the complete growth medium in each well of the poly-HEMA-coated well plate. The plate with seeded cells was centrifuged at 200 × g for 5 min and then placed on a rotary shaker (100 rpm) in a cell culture incubator (37°C, 5% CO_2_, 95% relative humidity). Spheroids were incubated without medium replacement on a rotary shaker (100 rpm) for 7 days. Three biological replicates of TMZ-treated and proliferating spheres were prepared. Several spheres from each replicate were used to assess viability and the proliferation index.

***Assessment of proliferation index by immunofluorescence staining of spheroid sections***

For immunofluorescence analysis, spheroids were fixed in 4% formaldehyde for 1 h, washed 3× with PBS, and embedded in 2% low-gelling temperature agarose/PBS at 65°C. Cooled samples were then processed using an ASP200 tissue processor (Leica Microsystems) and subsequently embedded in paraffin wax using an EG1150 embedding station (Leica Microsystems). Paraffin sections (4 µm) were dewaxed in xylene (3 × 10 min) and serially hydrated in ethanol/water (100%, 96%, 70%, 50%, 0% for 3 min each). Antigen unmasking was performed by boiling the slides in 0.01 M sodium citrate buffer (pH 6) in an electric pressure pot for 12 min, followed by cooling for another 1 h at room temperature. After that, sections were blocked with 10% FBS for 30 min and stained with primary anti-Ki67 antibody (RRID:AB_442101, NCL-L-Ki67-MM1, Leica Biosystems, Nussloch, Germany) diluted in PBS (1:200) overnight at 4°C. After washing with PBS (3 × 5 min), sections were incubated with Alexa Fluor 647-conjugated secondary antibody (Thermo Fisher Scientific) diluted in PBS (1:1000) for 60 min at room temperature. PBS-washed samples (3 × 5 min) were then cover-slipped using a Mowiol 4-88 containing DAPI (1 µg/mL). Images were acquired by a Leica DM6000 fluorescence microscope and processed using Fiji/ImageJ. The percentage of Ki-67 positivity and nucleus size were quantified by the open-source platform QuPath^3^ (more than 2,000 cells were analyzed per sample).

***Live/Dead assay***

To visually distinguish live and dead cells forming spheroids, cells were stained with a mixture of two dyes, calcein-AM (Invitrogen/Thermo Fisher Scientific) and propidium iodide (PI; Invitrogen/Thermo Fisher Scientific). Firstly, a dye solution containing 8 µM calcein-AM and 60 µM PI in cell culture medium without phenol red was prepared. Then, half the volume of cell culture medium was replaced in each well with the dye solution containing calcein-AM to a final concentration of 4 µM calcein and 30 µM PI. After incubation for 1 h at 37 °C in an incubator (5% CO2, 95% relative humidity), half the volume of medium in each well was exchanged for fresh medium without phenol red. Immediately prior to the microscopic visualization, spheroids were transferred from the poly-HEMA-coated well plates into a μ-Slide 8-well (Ibidi, Germany). Labeled spheroids were captured by 10 × objective (HC PL APO 10x/0.40 CS2) of the Andor Dragonfly 503 spinning disk confocal microscope. Images were taken as z-stacks with a 3 µm step size to capture the entire spheroids. Live cells stained by calcein-AM emitted green fluorescence light while dead cells stained by PI emitted red fluorescence light. Calcein-AM was excited by 488 nm wavelength laser and viewed with 525/50 nm bandpass filter, PI was excited by 561 nm wavelength laser and viewed with 600/50 nm bandpass filter. From each biological replicate, four spheroids were stained for the analysis. The images were processed in Imaris software (Oxford Instruments, RRID:SCR_007370).

***Sample preparation for proteomic analysis***

24 proliferating and 48 senescent spheroids were used for proteomic analysis of one biological replicate. After 7 days of incubation, spheroids were pooled, the cultivation medium was aspirated, and two washes with PBS (500 µl of PBS) were performed. The last step was to aspirate as much PBS as possible and store the spheroids at -80℃ until the next processing step. U87 spheroids were lysed with 3% sodium deoxycholate (SDC) in 200 mM triethylammonium bromide (TEAB) and sonicated for 10 min. Lysed samples were then heated at 70°C for 5 min, sonicated for an additional 5 min, treated with benzonase at 37°C for 30 min, and centrifuged at 4,000 × g for 5 min. Resulting supernatants were transferred to clean tubes, and protein concentration was estimated using a microBCA assay (Thermo Fisher Scientific). Fifty µg of protein was reduced with 5 mM tris(2-carboxyethyl)phosphine hydrochloride (TCEP) at 37°C for 30 min and alkylated with 10 mM S-methyl methanethiosulfonate (MMTS) at 25°C for 10 min. Proteins were precipitated overnight by adding 5 volumes of prechilled acetone. Samples were centrifuged at 12,000 × g for 5 min and redissolved in 100 mM TEAB. Proteins were digested by a mixture of lysyl endopeptidase and trypsin (Promega) at 1:25 enzyme-to-protein ratio (w/w) at 37°C overnight. Peptides were labeled with 200 mg of tandem mass tag (TMT) isobaric labels (Thermo Fisher Scientific) (corresponding to the TMT concentration of app. 25 mM) at 25°C for 60 min. Labeling efficiency and normalization factors in the created multiplex were assessed using a 60-min DDA run. Afterwards, the samples were quenched with 50% hydroxylamine (0.2% final concentration) at 25°C for 15 min and mixed. Peptides were desalted using peptide desalting spin columns (Thermo Fisher Scientific) according to the manufacturer’s instructions.

***High pH reversed-phase fractionation***

TMT multiplex was redissolved in 2% acetonitrile (ACN)/10 mM ammonium formate (NH_4_FA). Peptides were separated using XBridge BEH column (C18, 2.5 µm, 2.1 µm × 150 mm) (Waters) using a linear gradient of mobile phase B (80% ACN/10 mM NH_4_FA) at a ﬂow rate of 0.3 mL/min. The gradient was running from 0% B to 2% B in 2 min, from 2% B to 20% B in 9 min, from 20% B to 50% B in 41 min, and finally from 50% B to 52% B in 5.5 min. The total gradient time was 57.5 min. Fractions were collected into a 96-well polypropylene plate (Agilent Technologies) at 45 s intervals, starting from 3.7 min to 57.7 min, yielding 72 fractions with a volume of 225 µL each. The collected fractions were subsequently combined into 24 fractions and evaporated to dryness.

***Liquid chromatography separation and mass spectrometry data acquisition***

LC-MS/MS analysis was performed on a Dionex UltiMate 3000 RSLCnano HPLC system coupled with an Orbitrap Exploris 480 mass spectrometer (both Thermo Fisher Scientific) through a NanoSpray Flex (NG) ion source. Concatenated fractions were redissolved in 2% ACN/0.1% trifluoroacetic acid (TFA) and injected onto a PepMap100 C18 trap column (3 µm, 100 Å, 75 µm × 20 mm) and a PepMap RSLC C18 analytical column (2 µm, 100 Å, 75 µm × 250 mm) (both Thermo Fisher Scientiﬁc) in two technical replicates. Peptides were first loaded onto the trap column at a flow rate of 5 µL/min in 2% ACN/0.1% TFA for 5 min. The separation was achieved using eluent A, consisting of 2% ACN /0.1% formic acid (FA), and eluent B, consisting of 80% ACN/0.1% FA. The gradient ran from 2% B to 34.5% B in 70 min, followed by 34.5% B to 45% B in 10 min and a 5-min washing with 90% B at 250 nL/min. The nanospray ionization source was operated in positive-ion mode at a spray voltage of 1.8 kV. Full-scan MS spectra were recorded in profile mode over a scan range of 350-1400 m/z, with a resolution of 60,000, an AGC target of 300%, and a maximum injection time of 50 ms. The FAIMS Pro Duo interface was operated under compensation voltage of -45V and -60V and at a gas flow rate of 4.6 L/min. The top 10 most intense precursors were isolated using an isolation width of 1.3 m/z, a purity threshold of 70%, and a dynamic exclusion of 17 sec, followed by fragmentation with a normalized collision energy of 37%. MS2 spectra were recorded with a resolution of 30,000, AGC target of 200%, and maximum injection time of 68 ms.

***Mass spectrometry data processing and analysis***

Raw files were searched in Proteome Discoverer (version 3.0, RRID:SCR_014477) using the Sequest HT^4^ search engine against the human protein database (UniProt RRID:SCR_002380 UP000005640, SwissProt and Trembl entries, accessed in August 2024). Trypsin/P was used as a cleavage enzyme with up to 2 missed cleavages allowed. Thiomethylation (C) and TMT6plex (K, N-terminus) were set as fixed modifications, while oxidation (M) and N-terminal acetylation, loss of methionine, or their combination were set as dynamic modifications. The precursor and fragment mass tolerances were set to 10 ppm and 0.02 Da, respectively. The target-decoy strategy to control peptide false discovery and identifications was validated by Percolator^5^ (RRID:SCR_005040) software. The false discovery rate was kept at 1%. Only unique peptides assigned to each protein group were considered for quantification. TMT reporter intensities were adjusted using the manufacturer-provided correction factors for the corresponding isotopic distributions. Protein intensities were first normalized by equalizing the total sum of the total reporter ion intensities in each channel. Technical replicates were subsequently averaged.

***Data analysis***

The analyses were performed using Python 3.9.18^6^ (RRID:SCR_008394) and R 4.3.1^7^ (RRID:SCR_001905). Data visualization was conducted in Python using Seaborn 0.13.0^8^ (RRID:SCR_018132) and Matplotlib 3.8.3^9^ (RRID:SCR_008624). Differential gene expression analysis was performed in R using the limma 3.56.2 package^10^ (RRID:SCR_010943) from Bioconductor (RRID:SCR_006442). Gene set enrichment analysis (GSEA) was performed in R using the clusterProfiler 4.8.3 package^11^ (RRID:SCR_016884) and in Python using the GSEApy^12^ (RRID:SCR_025803) library. Signaling pathway impact analysis (SPIA) was conducted in R using the SPIA 2.52.0 package^13^, also available through Bioconductor. Statistical computations, including descriptive statistics and correlation analyses were carried out using SciPy 1.12.0^14^ (RRID:SCR_008058) in Python. Cell–cell communication analysis in a single-cell dataset was performed using CellPhoneDB 5.0.1^15^ (RRID:SCR_017054) in Python, where a permutation test for statistical significance is implemented.

# Supplementary Tables

**Supplementary Table S2:** An overview of some significant interactions of SCs with surrounding clusters and their outcome.

| **Signal producer** | **Ligand** | **Dominant signal receiver** | **Receptor** | **Outcome** |
| --- | --- | --- | --- | --- |
| SCs | CXCL14 | TAM, dividing B cells, dividing neuron, B cells, microglia, SCs, endothelial | CXCR4 | CXCL14 promotes glioma cells invasion, growth, recurrence, and is associated with poor survival in GB^16^. |
| SCs | ANXA1 | TAM, microglia | FPR1 | ANXA1-FPR1 modulates the immune response and inhibition reduced tumor growth and aggressiveness in breast cancer^17^. |
| SCs | ENTPD1 | TAM, microglia | ADORA3 | ENTPD1-ADORA3 leads to metastasis, proliferation, and enhanced cancer cell survival in breast and prostate cancer^18^. |
| SCs | LGALS3 | TAM, microglia, protoplasmic astrocyte | MERTK | Inhibition of MERTK in GB reverses the immunosuppressive state, inhibits neoangiogenesis, induces tumor cell death, and prolongs survival in mice^19^. |
| Endothelial | CXCL10, CXCL11 | SCs | DPP4 | DPP4 post-translationally modifies the chemokines CXCL10/11 which are essential for recruiting immune cells into the TME, and their modification reduces immune cell infiltration^20^. DPP4 inhibition improves anti-tumor responses in models of hepatocellular carcinoma and breast cancer^21^. High DPP4 expression correlates with the pathological glioma grade and is associated with poor prognosis in low-grade gliomas^22^. |
| SCs | APLN | SCs, pericytes, protoplasmic astrocytes, radial glia | APLNR | APLN-APLNR interaction leads to increased invasiveness and angiogenesis in GB^23^. |
| SCs | VEGFA | Endothelial, TAM, microglia, radial glia, pericyte, SCs | NRP1 | VEGFA-NRP1 interaction is essential for stimulation of endothelial cell migration and angiogenesis^24^. |
| SCs | PTPRD | Endothelial, mixed progenitor/neuron, dividing progenitor | IL1RAP | PTPRD-IL1RAP promotes GB progression^25^. |
| SCs | THBS2 | TAM | CD36 | THBS2 is positively associated with EMT and chemoresistance in colorectal cancer^26^. |
| SCs | PLAU | TAM, microglia, endothelial | PLAUR | PLAU-PLAUR interaction activates inflammatory processes and tumor-related signaling pathways and is associated with poor prognosis in primary and recurrent gliomas^27^. |
| Dividing B cells, microglia, TAM | AREG | SCs | EGFR | The cell surface receptor EGFR is crucial for the immunopathobiology of HCMV, as it regulates viral latency and replication^28^, facilitates virus entry into monocytes, and induces a unique proinflammatory motile phenotype in the infected cell, promoting viral spread^29^ and glioma invasion^30^. |

# Supplementary references

**1.** Dimri GP, Lee X, Basile G, et al. A biomarker that identifies senescent human cells in culture and in aging skin in vivo. *Proc Natl Acad Sci U S A.* 1995; 92(20):9363-9367.

**2.** Schindelin J, Arganda-Carreras I, Frise E, et al. Fiji: an open-source platform for biological-image analysis. *Nat Methods.* 2012; 9(7):676-682.

**3.** Bankhead P, Loughrey MB, Fernandez JA, et al. QuPath: Open source software for digital pathology image analysis. *Scientific reports.* 2017; 7(1):16878.

**4.** Eng JK, McCormack AL, Yates JR. An approach to correlate tandem mass spectral data of peptides with amino acid sequences in a protein database. *Journal of the American Society for Mass Spectrometry.* 1994; 5(11):976-989.

**5.** Käll L, Canterbury JD, Weston J, Noble WS, MacCoss MJ. Semi-supervised learning for peptide identification from shotgun proteomics datasets. *Nat Methods.* 2007; 4(11):923-925.

**6.** Rossum GV, Drake FL. *Python 3 Reference Manual*: CreateSpace; 2009.

**7.** Team RC. R: A Language and Environment for Statistical Computing2023.

**8.** Waskom M. seaborn: statistical data visualization. *The Journal of Open Source Software.* 2021; 6:3021.

**9.** Hunter JD. Matplotlib: A 2D Graphics Environment. *Computing in Science & Engineering.* 2007; 9(3):90-95.

**10.** Ritchie ME, Phipson B, Wu D, et al. limma powers differential expression analyses for RNA-sequencing and microarray studies. *Nucleic Acids Res.* 2015; 43(7):e47.

**11.** Yu G, Wang LG, Han Y, He QY. clusterProfiler: an R package for comparing biological themes among gene clusters. *Omics : a journal of integrative biology.* 2012; 16(5):284-287.

**12.** Fang Z, Liu X, Peltz G. GSEApy: a comprehensive package for performing gene set enrichment analysis in Python. *Bioinformatics.* 2022; 39(1).

**13.** Tarca AL, Kathri P, Draghici S. SPIA: Signaling Pathway Impact Analysis (SPIA) using combined evidence of pathway over-representation and unusual signaling perturbations2023.

**14.** Virtanen P, Gommers R, Oliphant TE, et al. SciPy 1.0: fundamental algorithms for scientific computing in Python. *Nat Methods.* 2020; 17(3):261-272.

**15.** Troulé K, Petryszak R, Cakir B, et al. CellPhoneDB v5: inferring cell-cell communication from single-cell multiomics data. *Nat Protoc.* 2025; 20(12):3412-3440.

**16.** Fazi B, Proserpio C, Galardi S, et al. The Expression of the Chemokine CXCL14 Correlates with Several Aggressive Aspects of Glioblastoma and Promotes Key Properties of Glioblastoma Cells. *International journal of molecular sciences.* 2019; 20(10).

**17.** Vecchi L, Alves Pereira Zóia M, Goss Santos T, et al. Inhibition of the AnxA1/FPR1 autocrine axis reduces MDA-MB-231 breast cancer cell growth and aggressiveness in vitro and in vivo. *Biochimica et biophysica acta. Molecular cell research.* 2018; 1865(9):1368-1382.

**18.** Shropshire DB, Acosta FM, Fang K, et al. Association of adenosine signaling gene signature with estrogen receptor-positive breast and prostate cancer bone metastasis. *Frontiers in medicine.* 2022; 9:965429.

**19.** Su YT, Butler M, Zhang M, et al. MerTK inhibition decreases immune suppressive glioblastoma-associated macrophages and neoangiogenesis in glioblastoma microenvironment. *Neuro-oncology advances.* 2020; 2(1):vdaa065.

**20.** Barreira da Silva R, Laird ME, Yatim N, Fiette L, Ingersoll MA, Albert ML. Dipeptidylpeptidase 4 inhibition enhances lymphocyte trafficking, improving both naturally occurring tumor immunity and immunotherapy. *Nat Immunol.* 2015; 16(8):850-858.

**21.** Hollande C, Boussier J, Ziai J, et al. Inhibition of the dipeptidyl peptidase DPP4 (CD26) reveals IL-33-dependent eosinophil-mediated control of tumor growth. *Nat Immunol.* 2019; 20(3):257-264.

**22.** Han Y, Sun Y, Zhang Y, Xia Q. High DPP4 expression predicts poor prognosis in patients with low-grade glioma. *Mol Biol Rep.* 2020; 47(3):2189-2196.

**23.** Kalin RE, Glass R. APLN/APLNR Signaling Controls Key Pathological Parameters of Glioblastoma. *Cancers.* 2021; 13(15).

**24.** Herzog B, Pellet-Many C, Britton G, Hartzoulakis B, Zachary IC. VEGF binding to NRP1 is essential for VEGF stimulation of endothelial cell migration, complex formation between NRP1 and VEGFR2, and signaling via FAK Tyr407 phosphorylation. *Mol Biol Cell.* 2011; 22(15):2766-2776.

**25.** Li F, Zhang W, Wang M, Jia P. IL1RAP regulated by PRPRD promotes gliomas progression via inducing neuronal synapse development and neuron differentiation in vitro. *Pathol Res Pract.* 2020; 216(11):153141.

**26.** Zhou X, Han J, Zuo A, et al. THBS2 + cancer-associated fibroblasts promote EMT leading to oxaliplatin resistance via COL8A1-mediated PI3K/AKT activation in colorectal cancer. *Mol Cancer.* 2024; 23(1):282.

**27.** Li J, Fan H, Zhou X, Xiang Y, Liu Y. Prognostic Significance and Gene Co-Expression Network of PLAU and PLAUR in Gliomas. *Frontiers in oncology.* 2021; 11:602321.

**28.** Buehler J, Zeltzer S, Reitsma J, et al. Opposing Regulation of the EGF Receptor: A Molecular Switch Controlling Cytomegalovirus Latency and Replication. *PLoS Pathog.* 2016; 12(5):e1005655.

**29.** Chan G, Nogalski MT, Yurochko AD. Activation of EGFR on monocytes is required for human cytomegalovirus entry and mediates cellular motility. *Proc Natl Acad Sci U S A.* 2009; 106(52):22369-22374.

**30.** Coniglio SJ, Eugenin E, Dobrenis K, et al. Microglial stimulation of glioblastoma invasion involves epidermal growth factor receptor (EGFR) and colony stimulating factor 1 receptor (CSF-1R) signaling. *Mol Med.* 2012; 18:519-527.

# Supplementary figure legends

**Supplementary Figure S1**: (**A**) Visualization of single-cell data using t-SNE showing individual cell clusters. (**B**) Cells that best match ‘common’ senescence markers in their expression. (**C**) Cells that predominantly express ECM (red), SASP (blue), and both ECM and SASP (green). (**D**) Overlap of genes from SASP and ECM sets, where only a small fraction (10%) of genes is shared. (**E**) Visualization of considerable overlap of cells with the highest expression of SASP and ECM components.

**Supplementary Figure S2**: Visualization of proliferating cells expressing *MKI67* (green), cells expressing *p21* and SASP (blue), and cells that have senescent features (the expression of *p21* and SASP) and at the same time expressing proliferation marker *MKI67* (red).

**Supplementary Figure S3**: (**A**) Number of DEGs in GB and lower-grade gliomas, sorted by deregulation direction. (**B**) Overlap of DEGs in GB with lower-grade gliomas. (**C**) Heatmap with enrichment scores of KEGG pathways in GB and lower-grade gliomas. The displayed signaling pathways are significantly enriched only in GB and a significant enrichment (adjusted p-value < 0.05) is highlighted with a green box.

**Supplementary Figure S4**: Verification of senescent phenotype and viability after TMZ treatment in 2D U87 and spheroids. After 14 days of treatment with four doses of TMZ (for experimental setup see scheme (**A**)), senescence was assessed by SA-β-gal staining. Representative images of SA-β-gal-positive cells (blue) are shown in (**B**), and their percentages are shown in (**C**) after analyzing at least 80a cells per biological replicate. DNA replication was evaluated by EdU incorporation: EdU was added on day 14 of 2D culture and cells were incubated for another 24 hours. Representative images of EdU-positive cells (red) detected by Click-chemistry, counterstained with DAPI and imaged by widefield high-content microscopy are shown in (**D**). The proportion of EdU-positive cells was quantified using ScanR analysis software across all three biological replicates (**E**). Because TMZ-treated 2D U87 cultures contained a subset of EdU-positive cells, we further characterized this population by measuring the nuclear size of these cells. The gallery of representative nuclei (**F**) and a corresponding column graph (**G**) show that EdU-positive nuclei in TMZ-treated cells are significantly larger than those in proliferating U87 cells. To verify the preservation of the senescent-like phenotype in 3D cell culture, paraffin sections from spheroids were stained for the proliferation marker Ki67 (red) and counterstained with DAPI (blue) (**H**). Quantification of Ki67-positive cells (**I**) and DAPI-labeled nuclear area (**J**) confirmed decreased cell proliferation and increased nuclear size in spheroids formed from cells pretreated with TMZ. Representative images indicating the viability of proliferating and senescent spheroids, estimated by calcein (live cells; green) and PI (dead cells; red), are shown (**K**). Statistical significance (**G**, **J**) among groups was analyzed by Mann–Whitney U test and significant changes are indicated by asterisks (****P < 0.0001). Scale bars: 20 μm (**F**); 100 μm (**B**, **D** and **H**); 200 μm (**K**).
