# Supplementary figures and images for: Cellular senescence escape and antiviral response discriminate glioblastoma from lower-grade gliomas
Source: Neurooncol Adv. 2026 Apr 8;8(1):vdag122. doi: 10.1093/noajnl/vdag122 (PMC13220956; doi:10.1093/noajnl/vdag122)

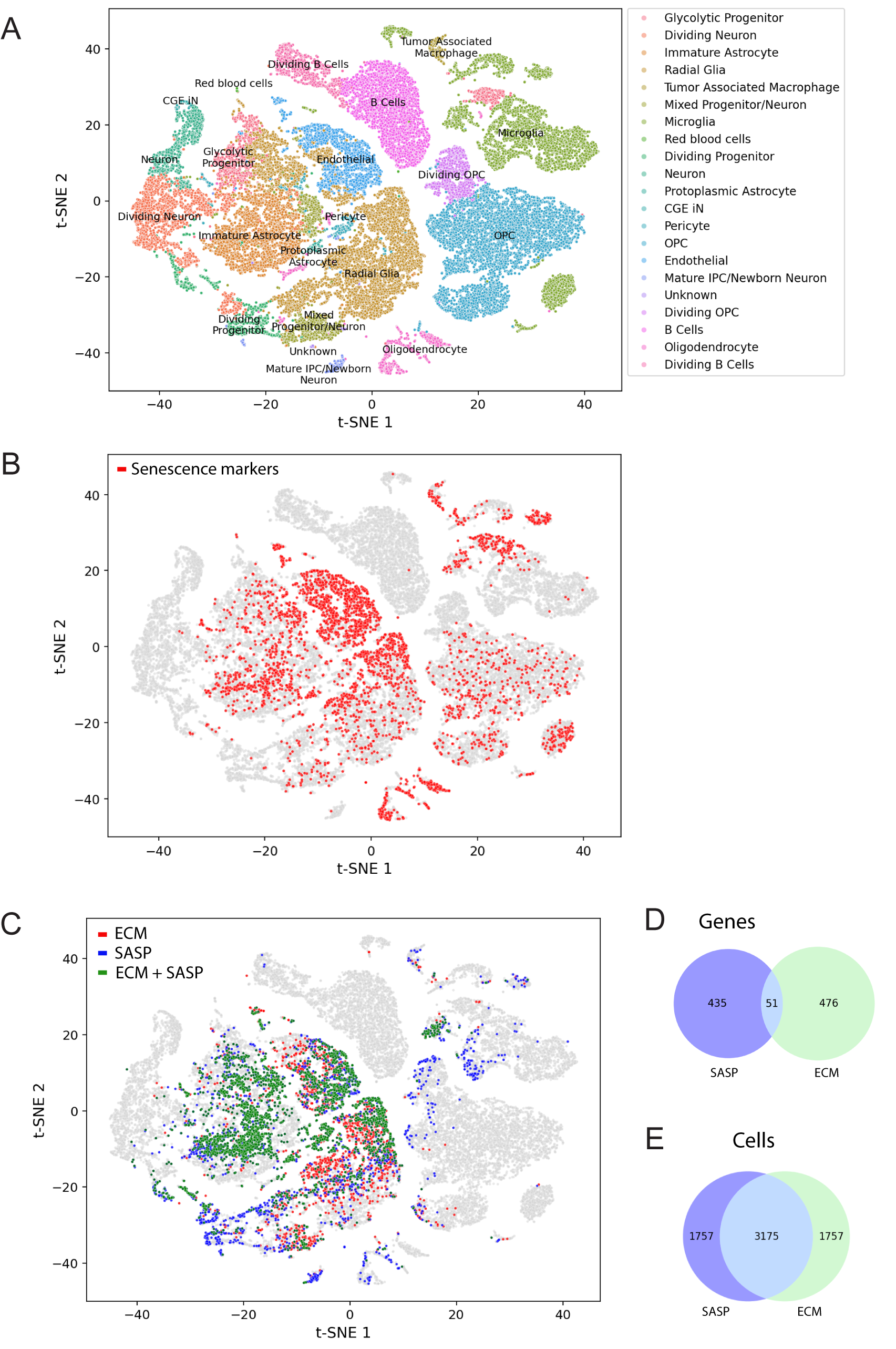

Supplement: vdag122_Supplementary_Data [file vdag122_supplementary_data.zip › Supplementary_Data (2)/Supplementary Figure S1.tif]

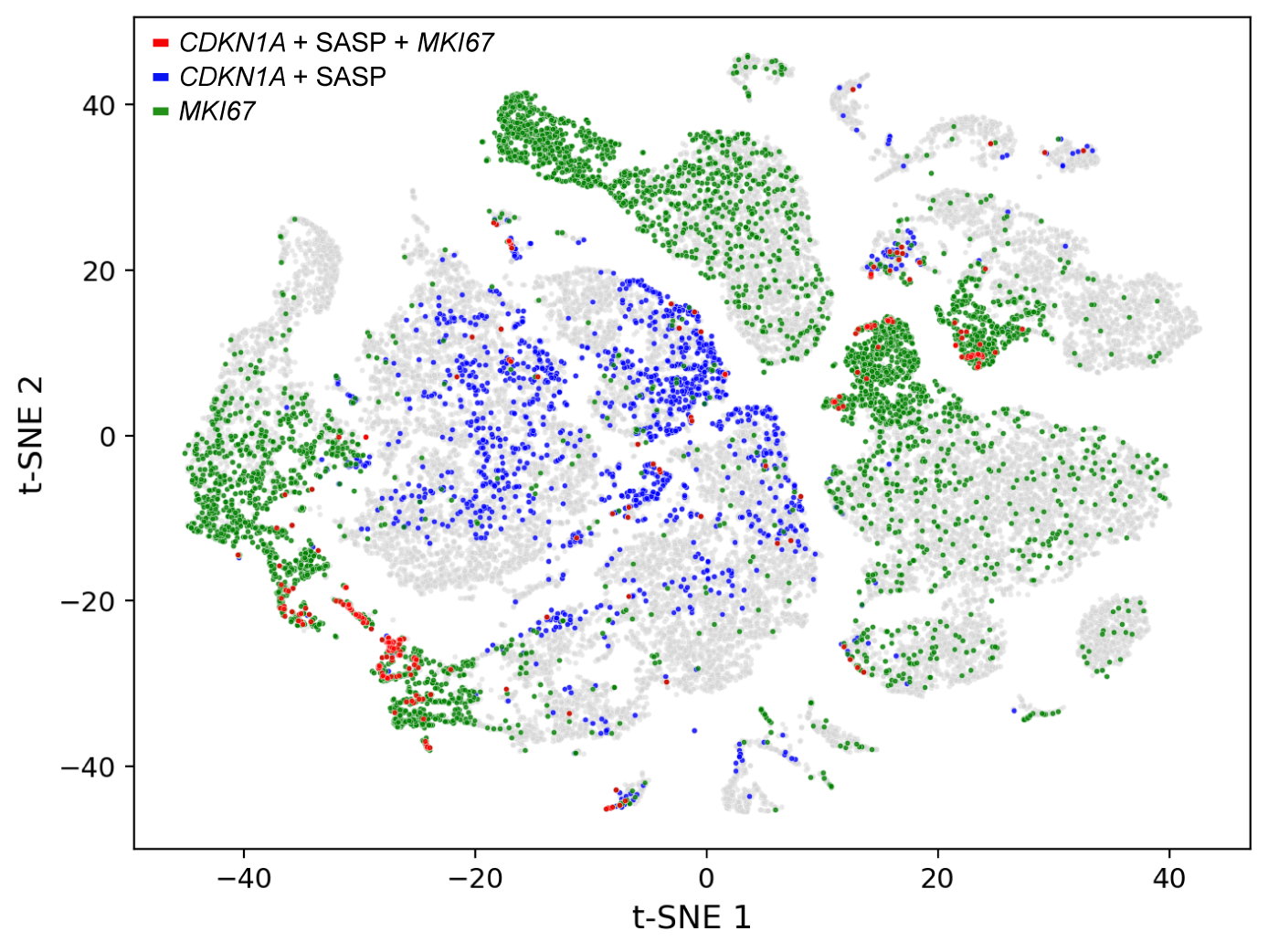

Supplement: vdag122_Supplementary_Data [file vdag122_supplementary_data.zip › Supplementary_Data (2)/Supplementary Figure S2.tif]

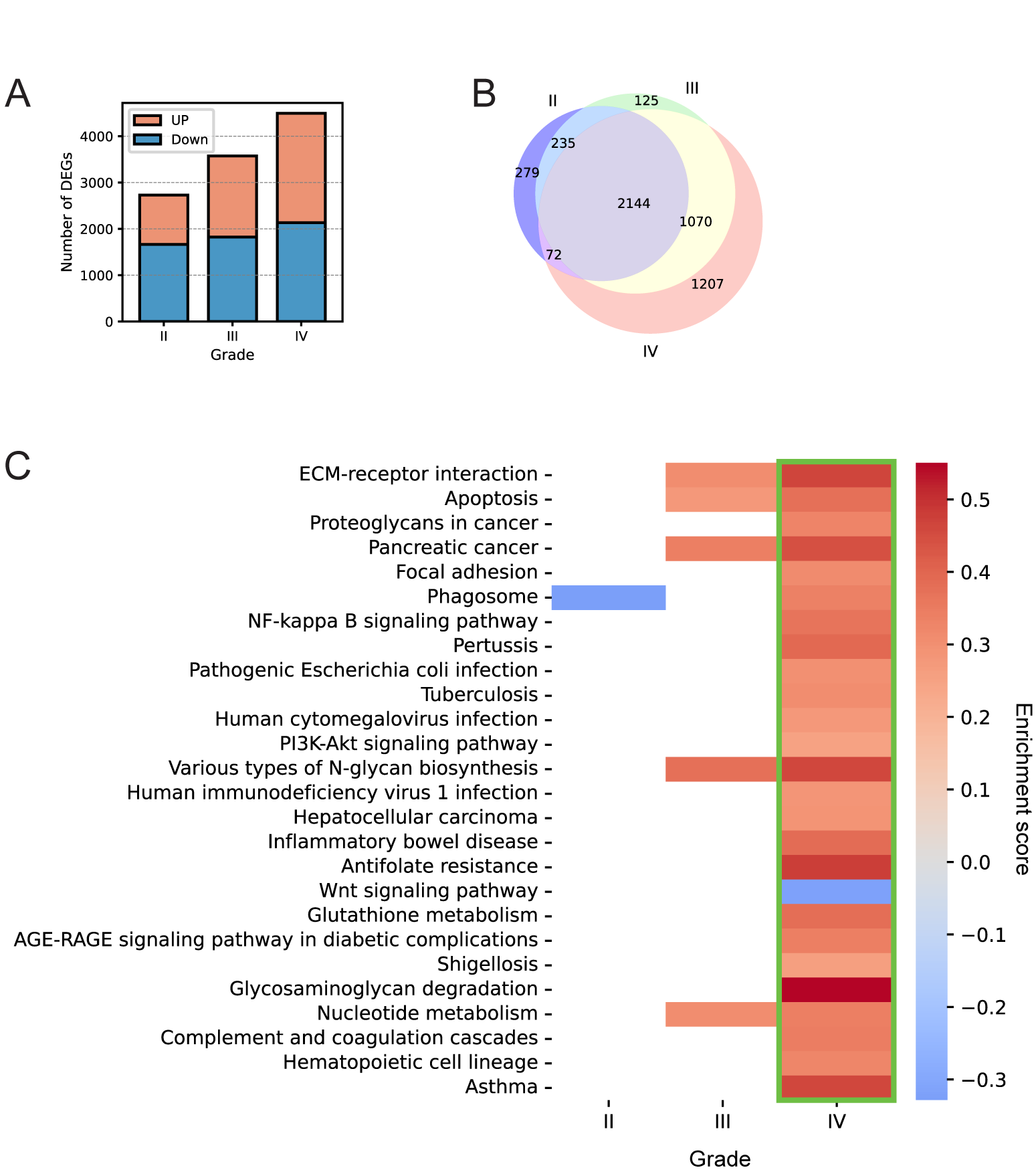

Supplement: vdag122_Supplementary_Data [file vdag122_supplementary_data.zip › Supplementary_Data (2)/Supplementary Figure S3.tif]

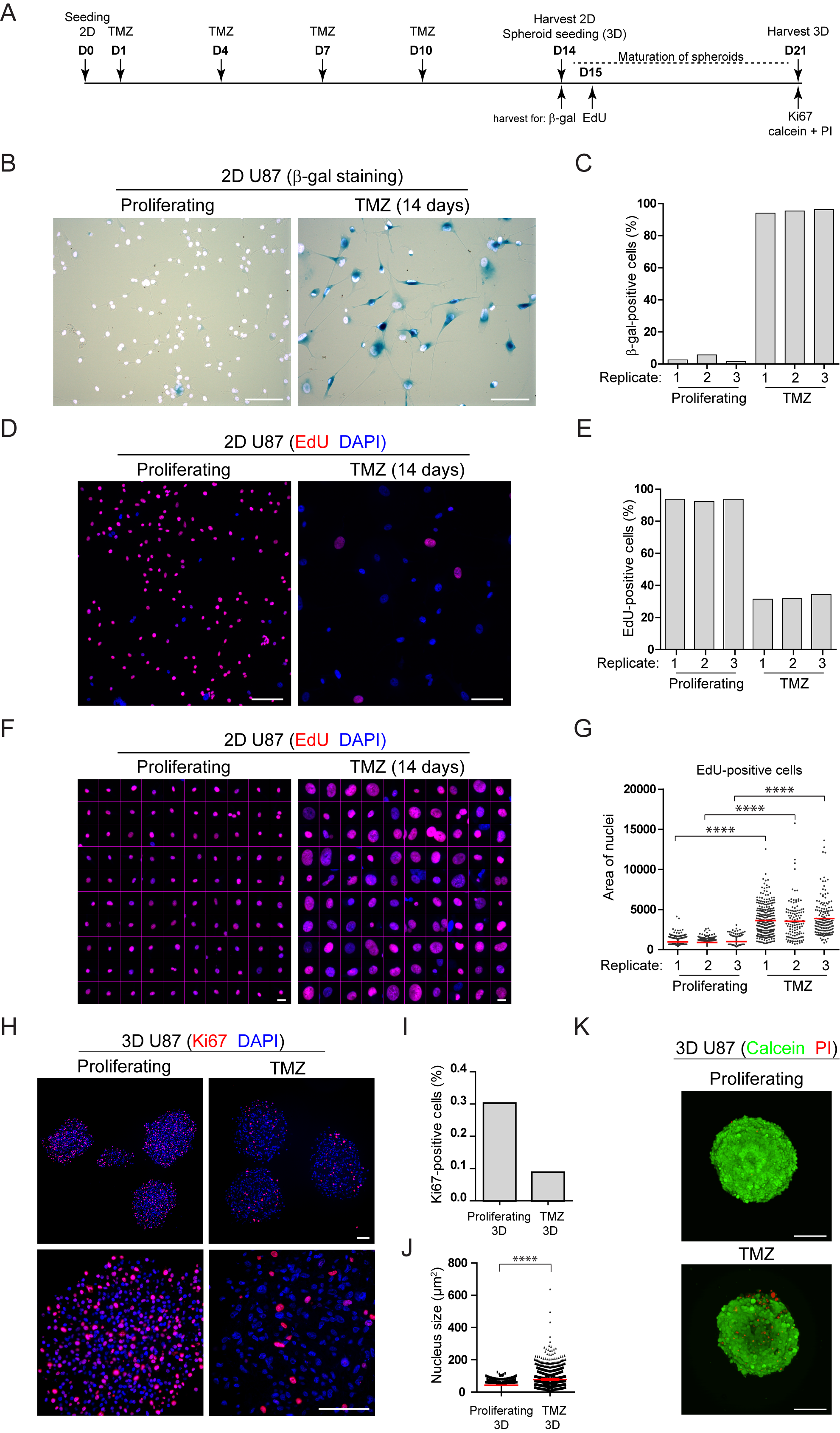

Supplement: vdag122_Supplementary_Data [file vdag122_supplementary_data.zip › Supplementary_Data (2)/Supplementary Figure S4.tif]
